# Neurocranial anatomy of the petalichthyid placoderm *Shearsbyaspis oepiki* Young revealed by X‐ray computed microtomography

**DOI:** 10.1111/pala.12345

**Published:** 2018-02-05

**Authors:** Marco Castiello, Martin D. Brazeau

**Affiliations:** ^1^ Department of Life Sciences Imperial College London Silwood Campus, Buckhurst Road Ascot SL5 7PY UK; ^2^ Department of Earth Sciences Natural History Museum London SW7 5BD UK

**Keywords:** petalichthyid, neurocranium, placoderms, computed tomography, stem gnathostomes, jawless vertebrates

## Abstract

Stem‐group gnathostomes reveal the sequence of character acquisition in the origin of modern jawed vertebrates. The petalichthyids are placoderm‐grade stem‐group gnathostomes known from both isolated skeletal material and rarer articulated specimens of one genus. They are of particular interest because of anatomical resemblances with osteostracans, the jawless sister group of jawed vertebrates. Because of this, they have become central to debates on the relationships of placoderms and the primitive cranial architecture of gnathostomes. However, among petalichthyids, only the braincase of *Macropetalichthys* has been studied in detail, and the diversity of neurocranial morphology in this group remains poorly documented. Using X‐ray computed microtomography, we investigated the endocranial morphology of *Shearsbyaspis oepiki* Young, a three‐dimensionally preserved petalichthyid from the Early Devonian of Taemas‐Wee Jasper, Australia. We generated virtual reconstructions of the external endocranial surfaces, orbital walls and cranial endocavity, including canals for major nerves and blood vessels. The neurocranium of *Shearsbyaspis* resembles that of *Macropetalichthys*, particularly in the morphology of the brain cavity, nerves and blood vessels. Many characters, including the morphology of the pituitary vein canal and the course of the trigeminal nerve, recall the morphology of osteostracans. Additionally, the presence of a parasphenoid in *Shearsbyaspis* (previously not known with confidence outside of arthrodires and osteichthyans) raises some questions about current proposals of placoderm paraphyly. Our detailed description of this specimen adds to the known morphological diversity of petalichthyids, and invites critical reappraisal of the phylogenetic relationships of placoderms.

Placoderms are generally considered to be the outgroup of modern jawed vertebrates (Young 1986; Janvier 1996a, b; Goujet 2001; Goujet & Young 2004; Brazeau 2009) and thus illuminate key phases in the stepwise evolution of jawed vertebrate anatomy. Placoderms have received renewed attention owing to recent investigations casting doubt on their monophyly (Friedman 2007; Brazeau 2009; Davis *et al*. 2012; Zhu *et al*. 2013; Long *et al*. 2015; *Zhu et al*. 2016). This debate impacts evolutionary questions ranging from the problem of morphological intermediates between jawless and jawed vertebrates to the evolution of vertebrate reproductive modes (Zhu *et al*. 2013, 2016; Brazeau & Friedman 2014; Long *et al*. 2015). In this paper, we add new observational data on the anatomy and diversity of placoderm cranial conditions and critically evaluate competing hypotheses of placoderm relationships in light of these data.

Of particular importance in phylogenetic studies of early vertebrates is the anatomy of the braincase. Our knowledge of placoderm endocranial anatomy is derived largely from the most diverse subgroup: the Arthrodira. Until recently, arthrodires were the predominant representatives of the placoderms, functioning as outgroup taxa in analyses of chondrichthyan (Coates & Sequeira 1998, 2001) and osteichthyan relationships (Zhu & Schultze 1997; Zhu *et al*. 1999, 2006, 2009). As arthrodire braincases are usually interpreted in light of crown gnathostome models, this potentially biases phylogenetic investigations (Brazeau & Friedman 2014). Since the works of Stensiö (1925, 1950, 1963a, b, 1969), only a few detailed descriptions of non‐arthrodiran braincases have been published (Ørvig 1975; Young 1978, 1980; Trinajstic *et al*. 2012; Dupret *et al*. 2017a). Many of these non‐arthrodiran taxa exhibit braincases departing significantly from an arthrodire ‘model’ and represent significant source of data on placoderm relationships.

Petalichthyida are non‐arthrodiran placoderms that have recently become central in the debate about placoderm relationships and primitive gnathostome anatomical conditions. This is due to perceived endocranial similarities with the jawless osteostracans and galeaspids (Janvier 1996a, b; Brazeau 2009; Brazeau & Friedman 2014). This has led to rejections of placoderm monophyly in numerical phylogenetic analyses, resulting in their recovery as a sub‐grade of stem‐group gnathostomes (Brazeau 2009; Davis *et al*. 2012; Zhu *et al*. 2013, 2016; Dupret *et al*. 2014; Giles *et al*. 2015).

Petalichthyida is presumed to be a monophyletic group, characterized by an X‐shaped sensory line pattern, two pairs of paranuchal plates and an elongated nuchal plate (Janvier 1996a). Within Petalichthyida, two divisions have been recognized: Macropetalichthyidae (Eastman 1898) and the Quasipetalichthyidae (Liu 1991). Along with these are forms like *Diandongpetalichthys* (P'an & Wang 1978), often recovered as sister to all other petalichthyids (Zhu 1991; Pan *et al*. 2015). Phylogenetic analyses suggest that quasipetalichthyids could either be sister group to the other petalichthyids (Zhu & Wang 1996; Pan *et al*. 2015) or fall in a more distant phylogenetic position from macropetalichthyids, closer to phyllolepid arthrodires (Giles *et al*. 2015). Therefore, some uncertainty about the monophyly of petalichthyids exists, though the latter analysis lacked many of the aforementioned petalichthyid apomorphies. Nevertheless, some of the characters used to define Petalichthyida, such as the X‐shaped sensory line pattern, are also present in other non‐petalichthyid placoderms (e.g. ptyctodonts) and might therefore be uninformative in characterizing petalichthyids. Furthermore, the phylogenetic position of the Petalichthyida within the stem gnathostomes is still disputed. Since some quasipetalichthyds, such as *Eurycaraspis*, have been used as examples of petalichthyids in recent analyses (Dupret *et al*. 2009, 2017b; Zhu *et al*. 2010), a better understanding of the systematics of the Petalichthyida will be crucial. A more detailed analysis of petalichthyid relationships is currently being undertaken by the authors.

Very few neurocranial remains are known for petalichthyids. The braincase of *Macropetalichthys*, a macropetalichthyid, currently being the most studied taxon. The first endocranial description of this placoderm was carried out by Stensiö (1925) and was based on a specimen from the Middle Devonian Onondaga Formation, New York. In this original description, the endocranial surface was exposed by fracturing the specimen with a hammer. Stensiö (1963a,b, 1969) later revised this reconstruction based on a mechanically prepared specimen, offering a more complete description of the endocavity. However, no other comprehensive examinations of petalichthyid internal anatomy have been carried out since, meaning that our knowledge of the endocranial anatomy of this group relies almost solely on Stensiö's interpretations.

Here we present our investigation of the cranial anatomy of *Shearsbyaspis oepiki* Young, 1985, a petalichthyid placoderm from the Early Devonian of New South Wales, Australia. It was first described by Young (1985) on the basis of two specimens: an incomplete skull roof with endocranial ossification attached and an incomplete skull roof preserved in visceral view. The holotype of *Shearsbyaspis*, is notable for its three‐dimensional preservation and perichondrally ossified neurocranium. The specimen was initially prepared using acetic acid, which allowed Young (1985) to describe important details of the orbit and the anterolateral margin of the braincase. However, this has also eliminated much of the matrix infill, allowing unimpeded X‐ray computed tomography scanning. Our study reveals the external endocranial surface, the endocranial cavity, cranial nerve and blood vessel canals, as well as additional details of the dermal and oral skeleton. These results will help inform current debates on early jawed vertebrate anatomical conditions.

## Material and method

The following description of the neurocranium of *Shearsbyaspis oepiki* Young, 1985 is based on the holotype specimen (NHMUK P33580), an incomplete skull roof with endocranial ossification. The specimen originates from the Shearsby's Wallpaper outcrop, in the *Spirifer yassensis* Limestone, the lowest unit of the Taemas Formation in the Murrumbidgee Group in New South Wales, Australia, dated to the Early Devonian (Emsian; Young 1978, 1980, 1985).

The specimen was scanned at the Imaging and Analysis Center at the Natural History Museum in London, using a Nikon Metrology HMX ST 225 X‐ray micro‐CT scanner. Scanning parameters were: 180 kV; 180 A; 0.1 mm thick copper filter; 6284 projections and voxel size 15.3 μm. The resulting tomographic data was segmented and rendered in 3D using the software Mimics 15.01 (Materialise Technologielaan, Leuven, Belgium). The 3D models produced with Mimics were imported in Blender (https://www.blender.org) for scientific image preparation (following Garwood & Dunlop 2014).

### Institutional abbreviations

CPC, Commonwealth Palaeontological Collection, Canberra, Australia; NHMUK, Natural History Museum, London, UK.

## Description

### Skull roof

#### Preservation and general features

**Figure 1:**
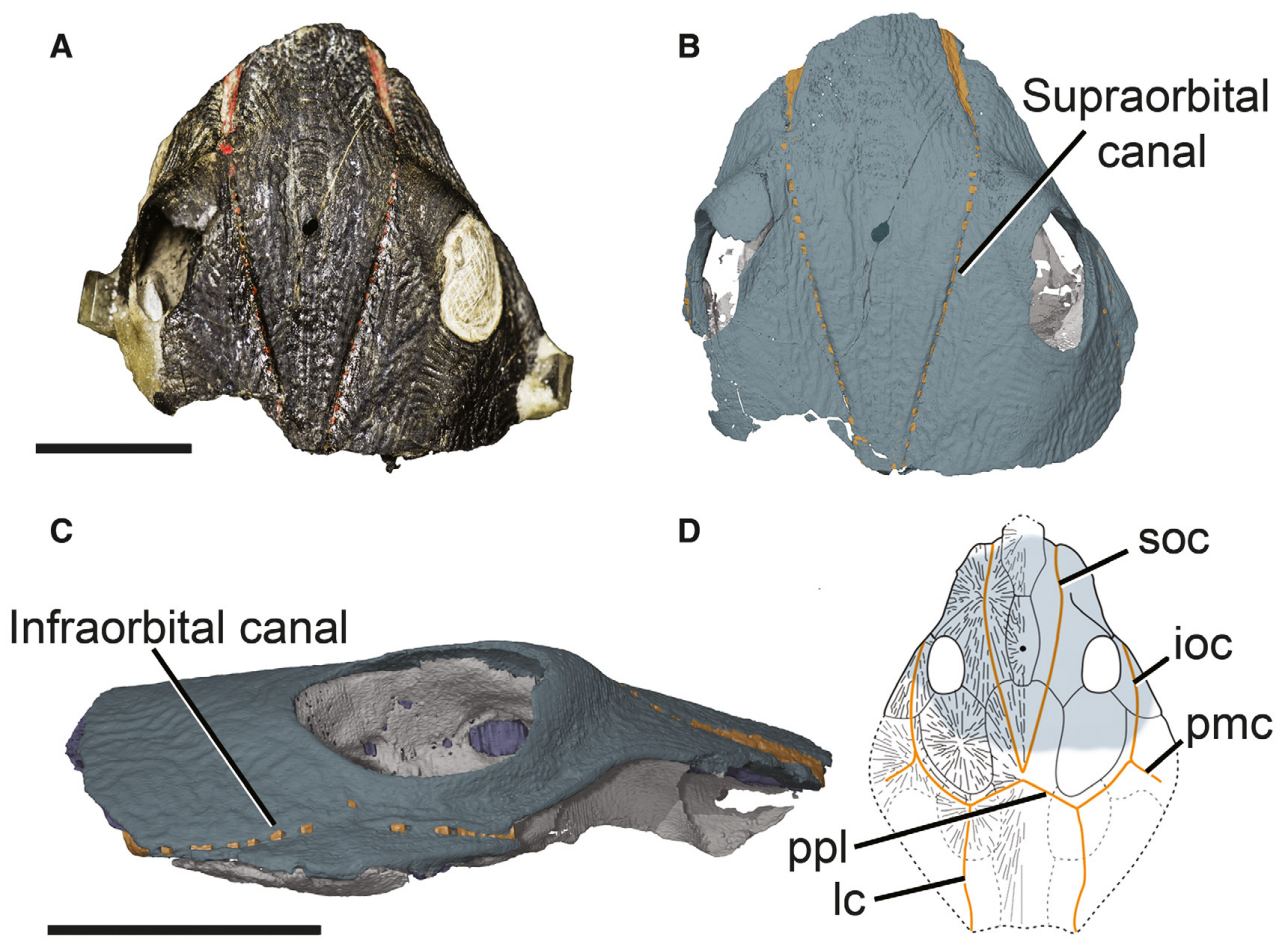
Skull roof and sensory lines of NHMUK P33580. A, photograph in dorsal view. B–C, 3D model in: B, dorsal; C, right lateral view. D, skull roof of *Shearsbyaspis*, redrawn after Young 1985, with the area preserved in NHMUK P33580 shaded. *Abbreviations*: ioc, infraorbital sensory canal; lc, main lateral line sensory canal; pmc, postmarginal sensory canal; ppl, posterior pit line canal; soc, supraorbital sensory canal. Scale bars represent 10 mm. Colour online.

NHMUK P33580 consists of an incomplete skull with most of the underlying neurocranial ossification preserved. The tip of the rostral plate and the remainder of the skull posterior to the otic region are missing. The dorsal side of the skull roof is exposed and well‐preserved, showing clearly the pores of the lateral line system, the pineal foramen and the orbits (Fig. 1). The visceral surface of the skull roof is visible from the CT data, displaying the prominent ridges corresponding to the passages of the sensory canals.

**Figure 2:**
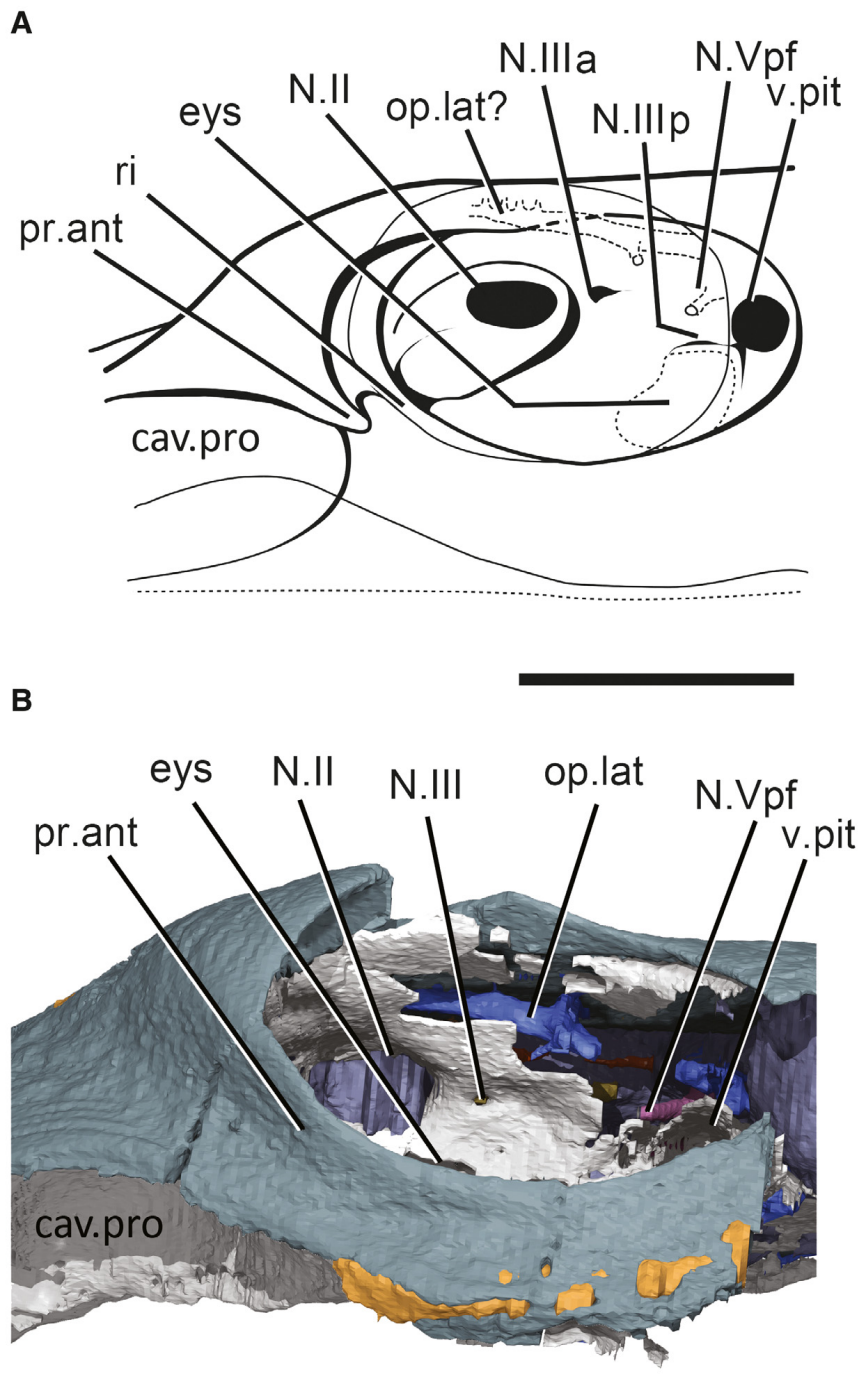
NHMUK P33580. A, left orbit redrawn after Young (1985). B, 3D model of the left orbit. *Abbreviations*: N.II, optic nerve foramen; N.III, oculomotor nerve foramen; N.IIIa, foramen for anterior branch of N.III; N.IIIp, foramen for posterior branch of N.III; N.Vpf, foramen for the profundus nerve; cav. pro, lateral preorbital space; eys, eyestalk attachment area; op.lat, canal for the ramus ophthalmicus superficialis lateralis; pr.ant, antorbital process; ri, ridge separating lateral preorbital space from orbital cavity; v.pit, foramen for pituitary vein. Colour online.

**Figure 3:**
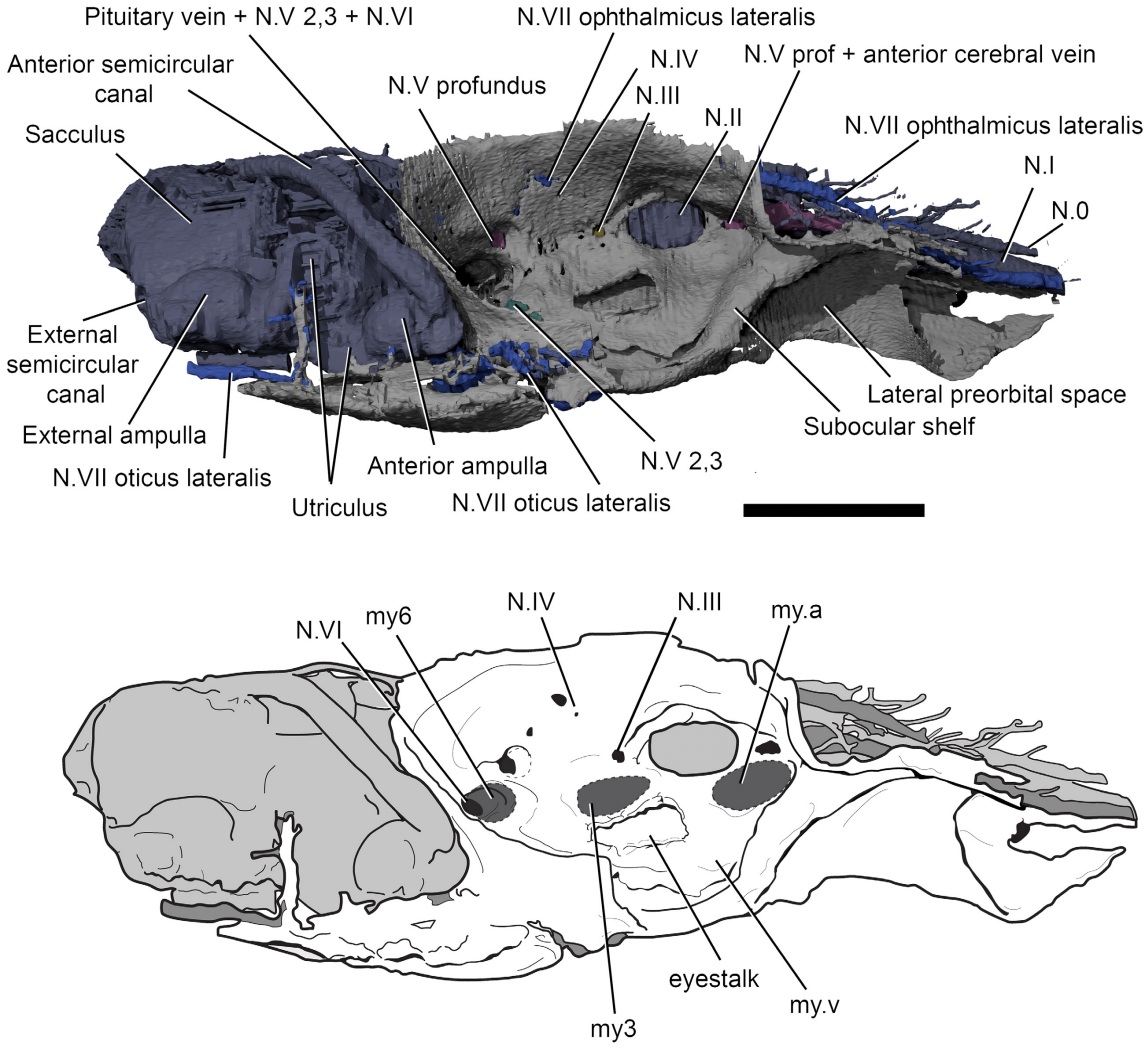
3D rendering (top) and line drawing (bottom) of right lateral surface of the braincase and endocavity of NHMUK P33580. *Abbreviations*: my3, myodome associated with cranial nerve III; my6, myodome associated with cranial nerve VI; my.a, anterior myodome; my.v, ventral myodome. Scale bar represents 5 mm.

**Figure 4:**
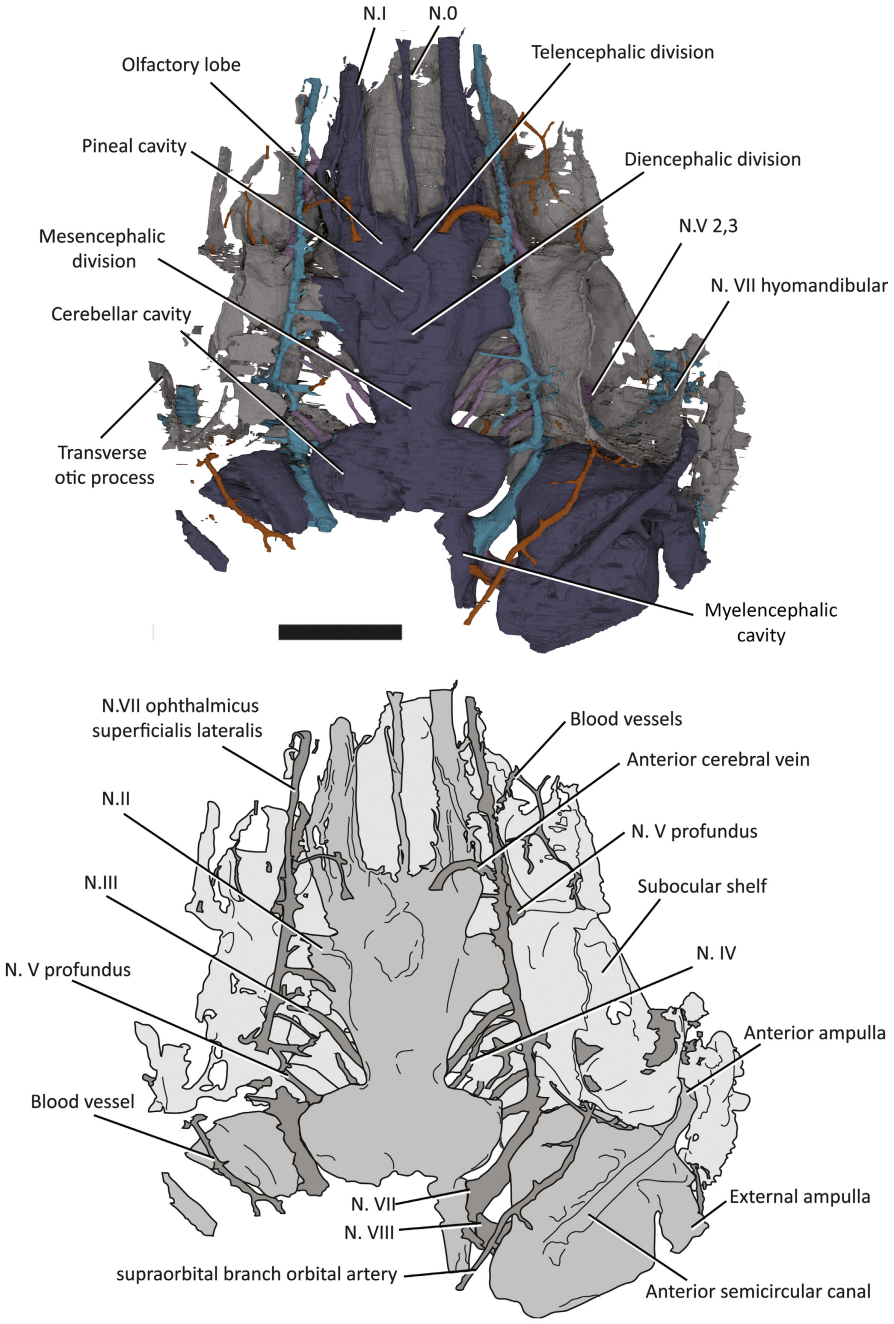
3D model (top) and line drawing (bottom) of the endocavity and braincase of NHMUK P33580, dorsal view. Scale bar represents 5 mm. Colour online.

The skull roof has a slight dorsally convex axial profile (Fig. 1). It reaches its maximum width at the level of the skeletal labyrinth, behind the posterior corner of the orbit. The lateral margins of the skull narrow anterior to the orbits, forming a broad, snout‐like projection. The orbits are dorsolaterally placed and display an anterodorsal torus, so that between the orbits the skull roof is slightly depressed (Figs 1, 2, 3, 4). The individual dermal plates are rarely distinct but some margins can be inferred from ornamentation patterns and radiating ridges corresponding to ossification centres. The dermal ornamentation consists of low concentric or radiating ridges, with rounded tubercles irregularly located along them.

The skull roof pattern (Fig. 1D) has already been described and figured by Young (1985) and we agree with his interpretations. Initially, the complete separation of the preorbitals by rostrals and pineal elements present in *Shearsbyaspis* was assigned as a diagnostic feature of this taxon. However, the same feature has since been observed in *Pampetalichthys longhuansis* (Zhu & Wang 1996; Zhu 2000). Nevertheless, *Shearsbyaspis* differs from *Pampetalichthys* in having anastomotic posterior pit line and supraorbital canals and cannot be considered synonyms.

#### Sensory line canals

**Figure 5:**
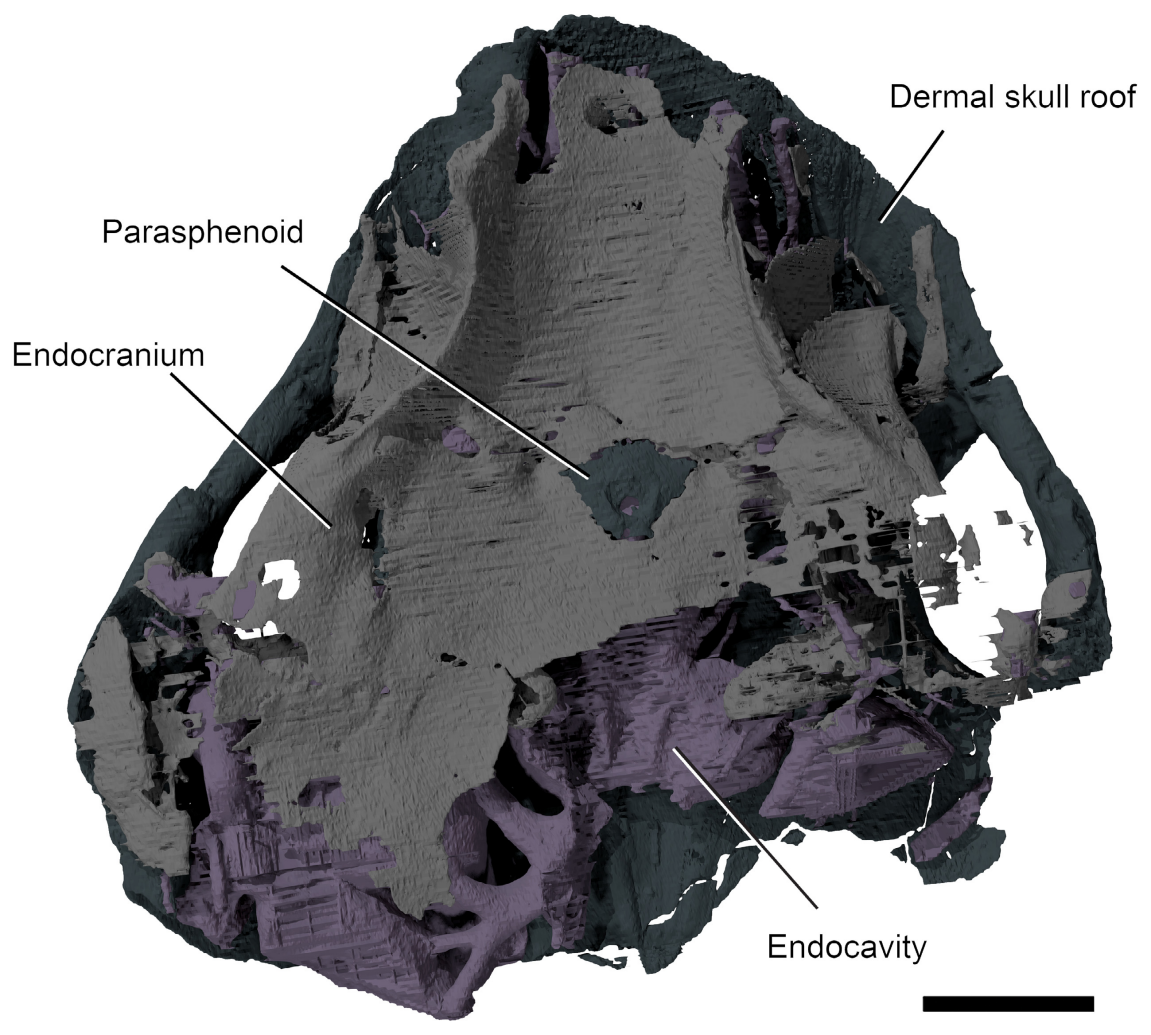
3D rendering of NHMUK P33580 in ventral view. Scale represents 5 mm. Colour online.

The sensory lines canals of NHMUK P33580 are well‐developed and clearly distinguishable on the dermal armour and illustrated in Figure 1D. They consist of enclosed tubes deeply situated in the bones, opening to the exterior through a single row of large pores, as in other petalichthyids and ptyctodontids (e.g. Stensiö 1925; Miles 1967; Denison 1978; Young 1978, 1985; Forey & Gardiner 1986; Zhu 1991; Trinajstic *et al*. 2012; Pan *et al*. 2015). Due to the incompleteness of the specimen, only the supraorbital sensory canals (soc) and part of the infraorbital sensory canals (ioc) are preserved in NHMUK P33580 (Fig. 1), but a complete restoration of the sensory line system of *Shearsbyaspis* can be made comparing both available specimens (NHMUK P33580 and CPC24622) as shown in Young (1985, text‐fig. 5).

The supraorbital sensory canal opens as pores or grooves depending on the position in the skull (Fig. 1B). In the rostral part of the skull, anterior to the eyes, each canal opens in a broad, deeply sunken groove. Nearer and posterior to the eyes they open as a row of distinct rounded and shallow pores. A similar condition is present in *Notopetalichthys* (Woodward 1941) and *Ellopetalichthys* (Ørvig 1957). There is a single enlarged pore opening marking the contact between the preorbital and nuchal plates as in the ptyctodontid *Materpiscis* (Trinajstic *et al*. 2012).

The infraorbital canal extends along the lateral margin of the postorbital plate and opens to the exterior as a single row of pores (Fig. 1C). The pores are more evenly spaced and sized in the posterior part of this sensory line, becoming a single narrow grove near the anterior part of the orbit. All the pore openings fall into a low, arcing line, except for a single pore situated as an outlier, nearer to the margin of the orbit (Fig. 1C). The tomographic image data show that this pore is associated with a set of numerous short and narrow canals connecting the orbit to the infraorbital canal, possibly representing a special sensorial area.

## Endocranium

### General features

**Figure 6:**
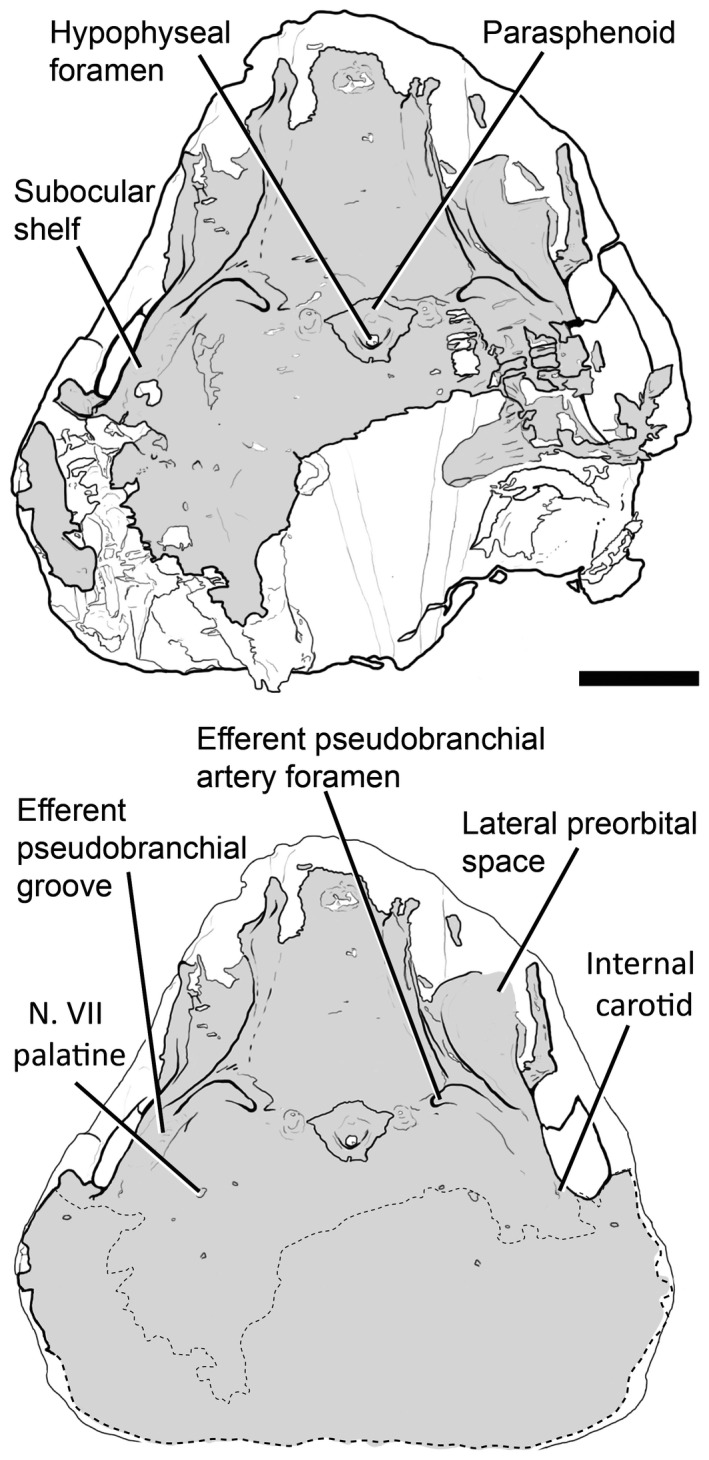
Drawing of the braincase of NHMUK P33580 in ventral view as preserved (top) and partly restored (bottom). Scale bar represents 5 mm.

In overall shape, the neurocranium is broad and flat, with a deeply concave ventral surface in transverse profile (Figs 3, 4, 5, 6). The braincase is surrounded by a thin layer of perichondral bone on the ventral and lateral side, while on the dorsal, a dorsal perichondral surface (if present) cannot be distinguished from the overlaying dermal bone. No evidence of an optic fissure is detected. The endocavity extends between the orbits, and an interorbital septum is not present, so that the braincase can be described as platybasic (Maisey 2007). In ventral view, the neurocranium is narrowest anteriorly, and broadens mediolaterally up to the lateral edges of the orbits (Figs 5, 6).

**Figure 7:**
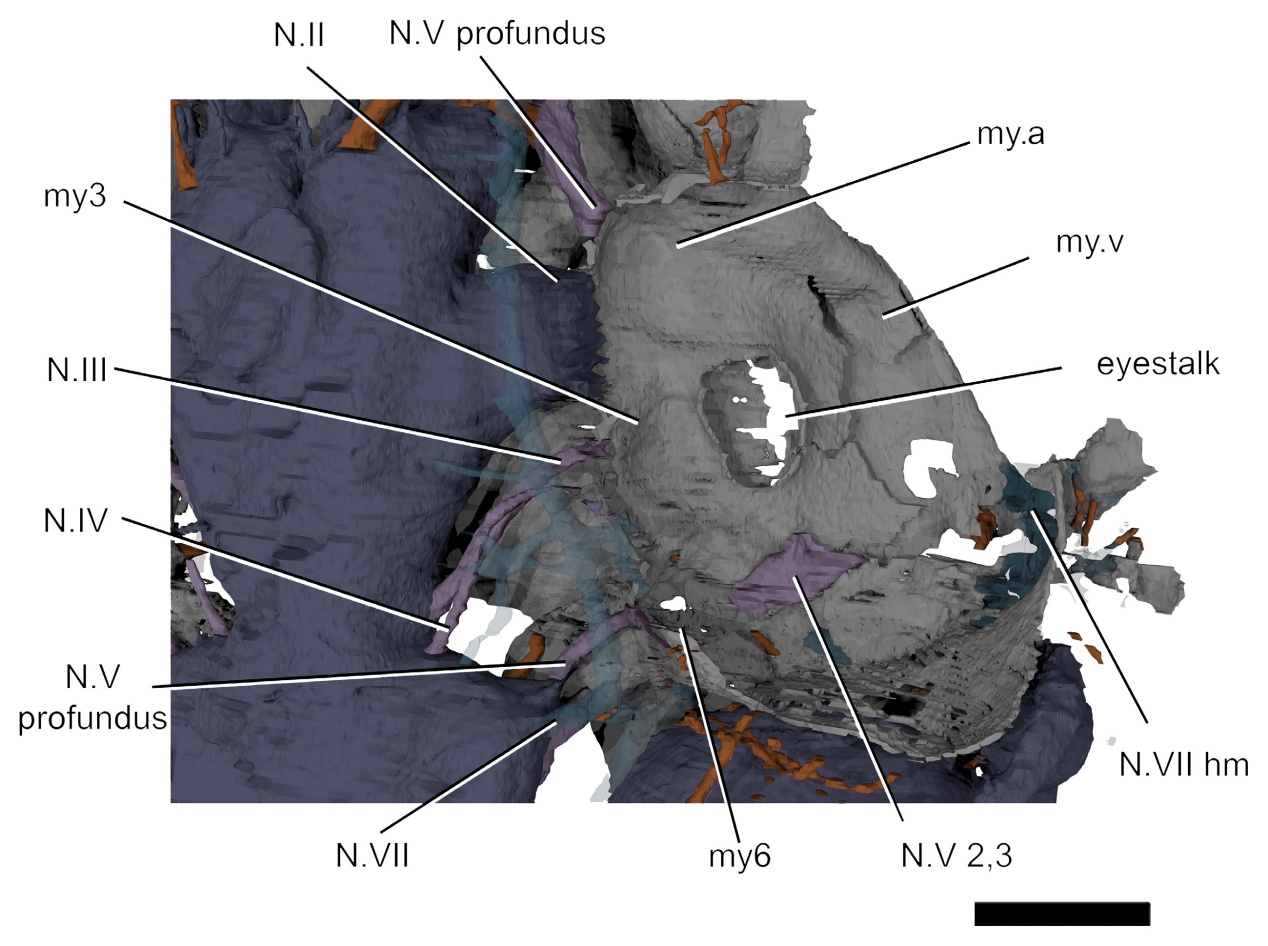
Internal view of the right orbit of *Shearsbyaspis* with dorsal part of the orbital wall removed to reveal the interpreted position of the extrinsic eye‐muscle myodomes and related innervations. *Abbreviations*: N.II, optic nerve canal; N.III oculomotor nerve canal; N.IV abducent nerve canal; N.V 2,3 trigeminal maxillary and mandibular canal; N.VII, main facial nerve canal; N.VII hm, canal for the hyomandibular branch of the facial; my.a, anterior myodome for an oculomotor‐innervated eye‐muscle; my.v, ventral myodome for an oculomotor‐innervated eye‐muscles; my3, myodome for an oculomotor‐innervated eye‐muscle; my6, myodome for abduscent‐innervated eye‐muscles. Scale bar represents 2.5 mm. Colour online.

On the ventral surface, grooves and foramina are observed indicating the passage of blood vessels and nerves (Figs 5, 6). On the ventral side of the subocular shelf, a series of grooves and foramina represent the course of the efferent pseudobranchial artery, the exit of the internal carotid, the palatine branch of the facial nerve (N.VII palatine) and other unidentified canals possibly representing blood vessels (Fig. 6). A parasphenoid is visible, surrounding the foramen for the hypophyseal duct. The parasphenoid is flanked by a pair of depressions (Figs 5, 6), similar to the hypophysial depressions in *Brindabellaspis* (Young 1980 text‐fig. 7). However, the depression on the anatomical left side is slightly obscured by breakage (Fig. 5).

### Orbital and lateral antorbital area

The left orbit is exposed and described by Young (1985). However, much of it is incompletely preserved (Fig. 2). The right orbit is still filled by matrix and thus relatively untouched by chemical or mechanical preparation (Figs 1C, 3), permitting a more complete description.

The orbits resemble *Macropetalichthys* in being set as dorsolaterally opening cavities (Figs 1, 2, 3, 4). The orbital cavity is bounded by anterior and posterior walls and a well‐developed subocular shelf (Figs 3, 4, 7). Therefore, contrary to the description of Young (1985), the preorbital space is not in communication with the orbit. Unlike in *Macropetalichthys* (Stensiö 1969), the lateral margin of the shelf is straight, rather than deeply embayed (Fig. 3). A large diameter opening for the optic tract (N.II) occurs in the anterior third of the medial wall of the orbit. The eyestalk attachment is present on the subocular shelf as an oval opening into the interperichondral space surrounded by an everted rim (Figs 3, 7). The position of the eyestalk attachment is here shown to be much more anterior and ventrolaterally placed relative to the optic nerve foramen than in Young's (1980) original interpretations (Figs 2, 3).

The arrangement of foramina and inferred myodomes are shown in Figures 3 and 7. Myodomes associated with cranial nerves III and VI (my3 and my6, respectively) are relatively unproblematic, resembling the disposition in other placoderms. Myodome 3 is situated immediately above the eyestalk attachment and immediately below the opening for the oculomotor nerve (N.III; Figs 3, 4, 5, 6, 7). The posteroventral myodome (my6) is developed as a deep, blind sac‐like pocket that accommodates openings for the trigeminal (N.V 2,3) and abducens (N.VI) nerves, and the pituitary vein (see endocavity description). This myodome has been interpreted as accommodating two rectus muscles in placoderms (Young 2008) and osteostracans (Janvier 1975).

Two additional putative myodomes are also observed on the orbital wall. There is a clear depression located immediately anterior to the optic tract (N.II) opening, which we consider to be an anterior myodome (my.a; corresponding to my_i_ of Stensiö 1969) possibly accommodating an oblique eye muscle. We cannot observe a ventral myodome (my.v) with confidence, as seen in many other placoderm taxa. This structure is normally located immediately below the eyestalk base. The corresponding area in *Shearsbyaspis* is located on the subocular shelf, which is broken on both sides of the specimen. However, a distinct pocket (Figs 3, 7, my.v) is visible near the anterolateral margin of the shelf. This myodome is commonly associated with the opening for the ophthalmica magna artery. Because the shelf is fragmented, we cannot verify this opening. However, a positionally correspondent opening is identified by Stensiö (1969 fig. 69) in *Macropetalichthys*, which he interpreted as accommodating the palatine branch of the facial nerve. However, we note that this canal communicates with the arterial canals in Stensiö's reconstruction.

A myodome associated with the troclear nerve (N.IV) is absent in *Shearsbyaspis* (Fig. 3). This nerve canal opens in the posterodorsal corner of the orbit, as in other placoderms. However, the surrounding perichondral surface is unimpressed by a myodome, as in arthrodires (Young 1978; Goujet 1984). Stensiö (1969, fig. 22B) reconstructed this nerve in *Macropetalichthys* in a more anterior position, confluent with the optic tract opening. This differs from the condition observed in *Shearsbyaspis* and all other placoderms. This therefore casts doubt on Stensiö's interpretation, and it is parsimonious to assume one of the more posterior openings documented in the orbit of *Macropetalichthys* corresponds to the trochlear nerve.

The profundus nerve opens posteroventrally to the trochlear nerve opening, at the same dorsoventral level as the optic tract opening (Fig. 3, N.V profundus). This opening occurs within a funnel‐shaped depression. While it is tempting to interpret this cavity as a myodome, we note that a similar cavity is observed around the profundus opening in other placoderms, such as *Buchanosteus* (Young 1979, figs 4, 8) and *Romundina* (Dupret *et al*. 2017a). On the dorsal surface of the subocular shelf, a wide groove extends anterorlaterally from the posteroventral myodome. This probably accommodated the mandibular and maxillary branches of the trigeminal nerve (N.V 2,3 Fig. 7). The course of the facial nerve (N.VII) is described later.

### Parasphenoid

**Figure 8:**
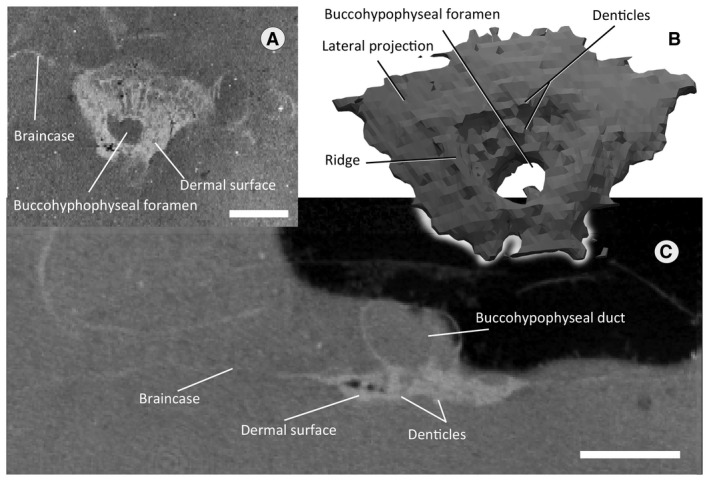
The parasphenoid of *Shearsbyaspis oepiki*. A, tomogram of the parasphenoid in coronal section. B, 3D rendering of the parasphenoid in ventral view. C, tomogram of the parasphenoid in axial section. Scale bar represents 2 mm.

At mid‐length along the basicranial surface, surrounding the hypophyseal opening is a distinct, spongiose dermal ossification applied to the perichondral bone which we identify as the parasphenoid bone (Fig. 8). The sub‐axial and sub‐coronal tomography orientations reveal that the bone has a clear separation from the perichondral bone and is thus a separate ossification (Fig. 8; Castiello & Brazeau 2017). The bone is as wide as it is long, flared anteriorly and tapering posteriorly (Fig. 8B), giving it a pentagonal shape. The buccohypophyseal foramen is a distinct, subcircular opening posterior to the centre of the parasphenoid. The opening is situated in a round depression surrounded by a raised ridge (Fig. 8B). The bone is thickest and spongiose near its geometrical centre, and thins out towards the margins (Fig. 8). The ventral (oral) surface of the bone bears irregularly sized protrusions which we interpret as denticles (Fig. 8B–C). They are unevenly distributed, and increasing in size near the buccohypophyseal foramen. The parasphenoid differs from arthrodires in lacking foramina or grooves for the paired hypophyseal vein (Stensiö 1969; Goujet 1984; Dupret 2010).

## Endocranial cavity

**Figure 9:**
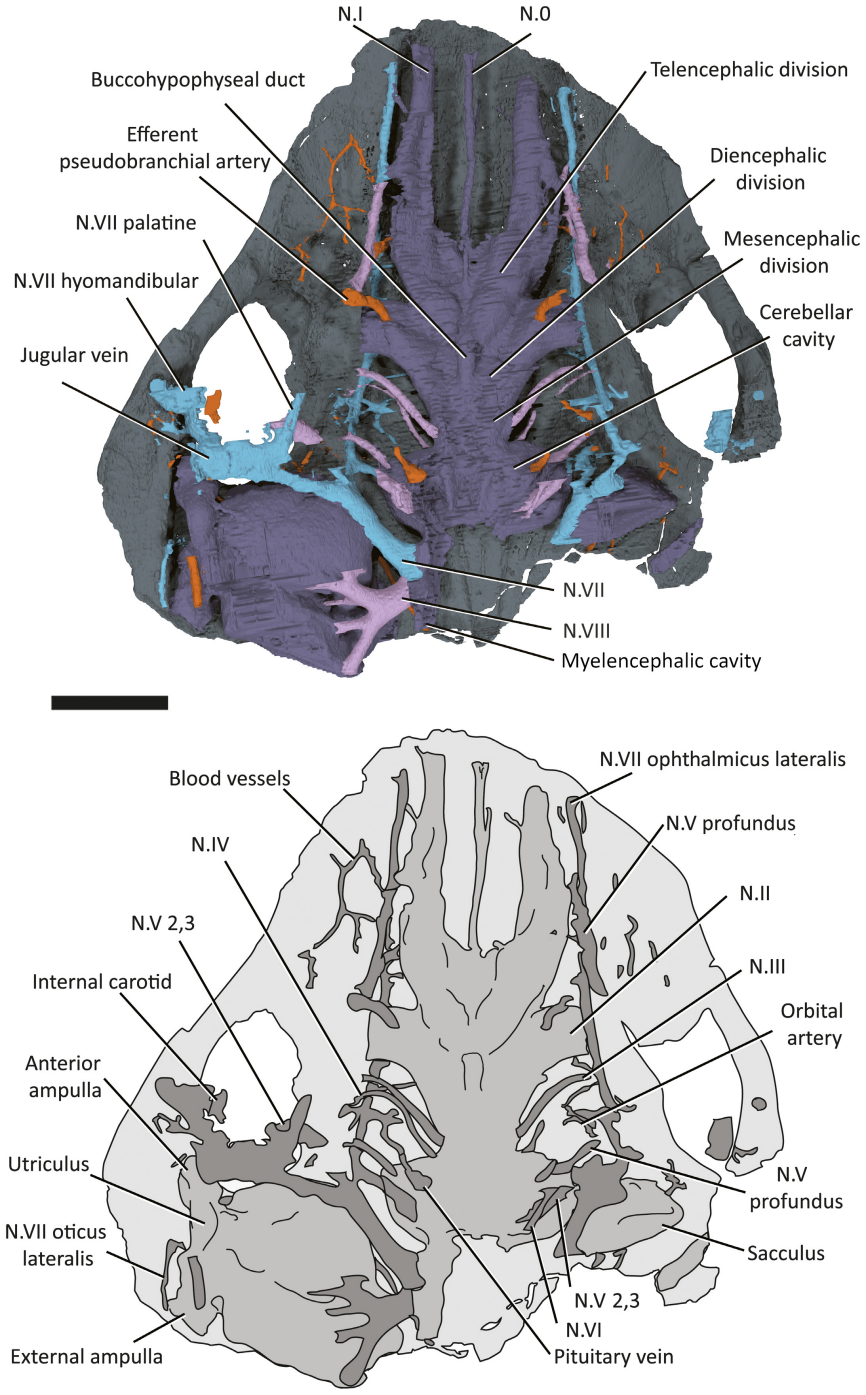
3D model (top) and line drawing (bottom) of the endocast of NHMUK P33580, ventral view. Scale bar represents 5 mm. Colour online.

The cranial endocast is well preserved, but incomplete posterior to the otic capsules (Figs 4, 9). It is long and narrow, with anteriorly directed elongated olfactory ducts, similar to *Macropetalichthys* (Stensiö 1925, 1963a, 1969). The endocavity of NHMUK P33580 resembles that of other placoderms, as well as many chondrichthyans in being situated directly between the orbits (platybasic) (Fig. 4).

The division between the telencephalon and diencephalon is most clearly visible on the ventral surface. There is a pair of well‐developed cavities, anterior to the optic tract canals, representing the olfactory lobes. The ventral portion of the olfactory lobes appears longer, extending posteriorly to level the hypophysial canal opening (Fig. 4).

The diencephalon cavity issues the optic nerve (N.II) canal, as well as the pineal cavity and the hypophysial cavities. In ventral view (Fig. 9), the diencephalon cavity of *Shearsbyaspis* differs from *Macropetalichthys* in two aspects. *Shearsbyaspis* lacks two well differentiated lobes in the hypothalamus cavity. Furthermore, the pineal cavity is lozenge‐shape and asymmetrical instead of tubular and wider at the base as described in *Macropetalichthys* (Stensiö 1969). Two ridges associated with the central body of the pineal gland could be related with the parietal organ. The pineal organ opens on the dermal skull through a foramen between the anterior parts of the orbits. The hypophyseal duct is placed slightly posteriorly in respect of the pineal gland and projects anteroventrally, opening through the palate with a single foramen in the parasphenoid (Figs 5, 6, 8).

The anterior limit of the midbrain cavity is not clear, but must be placed behind the optic nerve and the hypophyseal duct. In dorsal view, its posterior boundary is clearly marked by the sharp lateral expansion of the cerebellar cavity (Fig. 4). No distinct optic lobe cavities are present.

The hindbrain cavity is partially preserved as a complete metencephalon cavity and the partial right side of the myelencephalon cavity. The division between the metencephalic and the myelencephalic cavities is clearly marked by a constriction (visible in dorsal or ventral views) anterior to the trunk of the facial nerve canal (Figs 4, 9). The metencephalon is constituted by two symmetrical rounded lobes, probably representing the location of the cerebellum, while the myelencephalic portion is straight and narrow (Figs 4, 9).

## Cranial nerves canals

### Olfactory and terminal nerve canals

**Figure 10:**
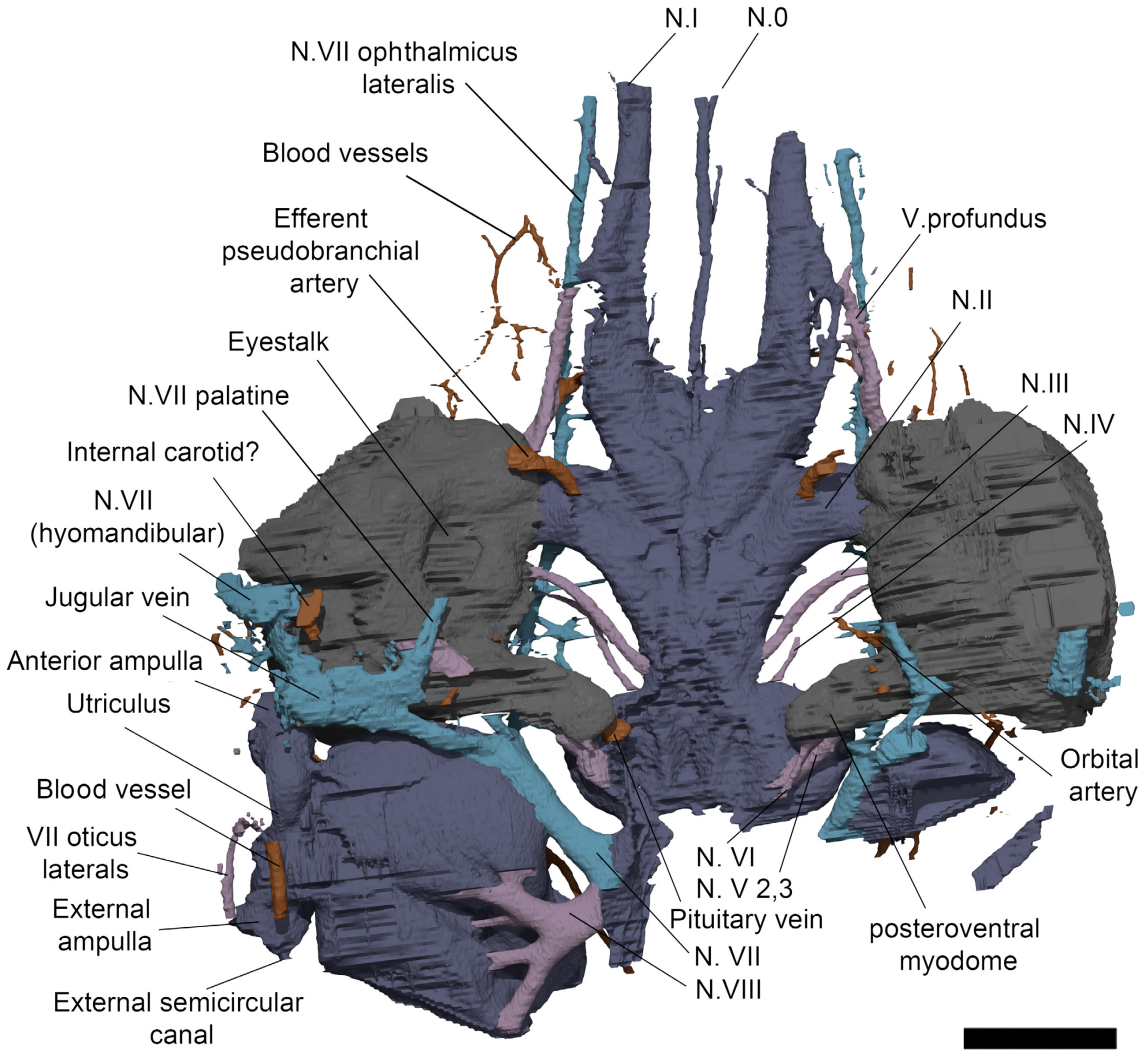
3D model of the endocast of NHMUK P33580 with the addition of the endocast of the orbital cavity, ventral view. Scale bar represents 5 mm. Colour online.

Two long, large‐diameter olfactory tract (N.I) canals extend from the olfactory lobes of the telencephalon to the anterior margin of the skull (Figs 4, 9, 10). The nasal capsules are not preserved, but considering the course of the olfactory nerve, they would have been situated at the anteriormost margin of the skull. An unpaired canal extending between the olfactory canals from the anterior ventral edge of the telencephalic, is interpreted as an unpaired terminal nerve (N.0) (Figs 4, 9, 10).

### Optic, oculomotor, trochlear and abducens nerve canals

The optic nerve (N.II) tract (Figs 4, 9, 10) arises from the diencephalic portion of the endocranial cavity (Figs 4, 9, 10). The oculomotor (N.III) canal (Figs 4, 9, 10) exits from the mesencephalic cavity and extends anterolaterally, paralleling the optic tract, to connect to the orbital wall. It opens posterior to the optic nerve foramen, anteriorly to the myodome for the internal rectus muscle (my3; Figs 3, 7). The trochlear (N.IV) nerve canal (Figs 4, 9, 10) extends from the anterior boundary of the cerebellar cavity, at the narrow waist between the optic lobe and cerebellum and parallels the course of the oculomotor canal. It meets the orbital wall and opens as a foramen located directly dorsal to the oculomotor foramen. The abducens (N.VI) nerve canal (Figs 9, 10) is a narrow‐diameter canal extending from the ventral surface of the cerebellar cavity to posteromedial corner of the posteroventral myodome (Fig. 10).

### Trigeminal nerve canals

There are two distinct canals accommodating branches of the trigeminal (N.V) (Figs 4, 9, 10), as opposed to a single, laterally projecting trigeminal trunk recess as in arthrodires (Stensiö 1963a, 1969; Young 1979; Goujet 1984). The profundus (V pf) canal originates separately from the common canal of the remaining branches. It extends from the dorsal anterior edge of the cerebellar cavity to a foramen situated above the posteroventral myodome (Figs 3, 4, 7). Unlike in other placoderms (e.g. in *Buchanosteus*, Young 1979, fig. 4) there is no groove on the orbital wall to distinguish its course. However, a canal exiting the anteromedial wall of the orbit and paralleling the course of the ophthalmic branch of the facial nerve (described below) can be interpreted as the continuation of the profundus nerve canal (Figs 3, 4, 7).

The remaining trigeminal nerves canals (N.V 2,3; Figs 9, 10) extend from the ventral side of the cerebellum to the posteroventral myodome, piercing its ventral wall. Here, it would have divided into the maxillary and mandibular branches. A broad groove exiting from the posteroventral myodome on the ventral side of the orbital wall (N.V 2,3; Figs 3, 4, 7) may have accommodated the course of these two branches (cf. *Romundina*, Dupret *et al*. 2014, 2017a).

### Facial nerve canal

The trunk of the facial nerve (N.VII) canal exits immediately anterior to the trunk of the acoustic nerve canal (see below), with the two canals (VII and VIII) being nearly confluent at the endocavity wall (Figs 4, 9, 10). A very short common canal for the ophthalmicus superficial lateralis and the ramus buccalis lateralis divides from the main facial nerve canal just medial to the level of the orbital wall (Figs 4, 9). A short canal for the ramus buccalis lateralis enters the orbit via the posterior wall of the posteroventral myodome (Figs 4, 9). The ophthalmic superficialis lateralis canal (N.VII oph. lat) extends dorsally from the main canal at a high angle before bending sharply towards the snout. It runs anteriorly between the orbital and endocranial cavities following the course of the supraorbital sensory canal (Figs 4, 9). The ophthalmic superficialis lateralis canal merges with the profundus nerve canals and the olfactory tracts as it extends towards the snout (Figs 4, 9).

The main body of the facial nerve passes behind the orbit towards the external (lateral) side of the braincase. It divides into the palatine branch (N.VII. palatine) and the hyomandibular branch (N.VII hyomandibular) (Figs 9, 10). The palatine branch passes beneath the orbit, remaining invested in the subocular shelf, and opens to the basicranium beneath the orbit. The hyomandibular branch enters the orbit, and continues as a sulcus in the posterior floor of the orbit before passing out the lateral side of the orbit as a short (partially preserved canal) in the transverse otic process (N.VII hm; Figs 3, 7). Several small canals, branching anterior to the anterior ampulla and ultimately connected with the infraorbital sensory canal. These are interpreted as canals for the ramus oticus lateralis (N.VII ot.lat.) (Figs 9, 10).

### Acoustic nerve

The acoustic (N.VIII) nerve innervated the labyrinth through an anterior and a posterior branch. Each branch further divided into two small canals before entering the ventromesial wall of the saccular chamber (Figs 9, 10).

## Vascular system canals

### Arteries

The course of the efferent pseudobranchial artery is indicated by a groove on the ventral side of the subocular shelf. It pierces the braincase next to the parasphenoid (Figs 5, 6, 9, 10) and enters the endocavity via the optic tract canal. A short canal pierces the base of the transverse otic process, and corresponds with the position of the orbital artery canal in *Buchanosteus* (Young 1979). Another pair of canals, extending above the sacculus and entering in the posterodorsal wall of the orbit (Fig. 10), are tentatively assigned to the supraorbital branches of the orbital artery (cf. *Brindabellaspis*; Young 1980, fig. 10).

### Veins

The pituitary vein in *Shearsbyaspis* is hypothesized to have entered the orbit via the posteroventral myodome as two separate canals (Figs 9, 10). Unlike other placoderms, there is no sign of the hypophysial vein. Only a short anterior segment of the jugular vein canal is preserved (Figs 9, 10). It opens at the back of the orbit, confluent with the facial nerve canal.

A pair of canals originates from the dorsal portion of the telencephalon and loop back toward the orbit, opening on the anterodorsal wall of the orbital cavity (Fig. 4). In shape, they are very similar to that which has been identified in *Brindabellaspis* as the terminal nerve (N.0). Here, however, the nasal capsules are located far anterior to the orbits so that these canals are more likely to have carried a blood vessel. A similar pair of canals, exiting from the telencephalic region and entering the anterior wall of the orbit, have instead been identified as the anterior cerebral vein in many placoderms (e.g. Dupret *et al*. 2014, 2017a,b; Young 1979) and osteostracans (e.g. Janvier 1985). A partial corresponding canal was figured in *Macropetalichthys* by Stensiö (1969), but was left unidentified.

## Labyrinth cavity

Parts of both the labyrinth cavity are preserved in NHMUK P33580 (Figs 3, 4, 9, 10). The right labyrinth preserves the position and morphology of the vestibule apparatus and related cranial nerves entering in it (Figs 3, 4, 9, 10). Only the anterior semicircular canal is completely preserved, and a short anterior segment of the horizontal canal is preserved. The saccular portion is deep and as large as, or larger, than the orbit (Fig. 4). The orbit and the labyrinth are almost in contact with one another (Figs 3, 4), so that the facial nerve passes so close to the labyrinth that it intersects the saccular wall (Figs 4, 9, 10).

**Figure 11:**
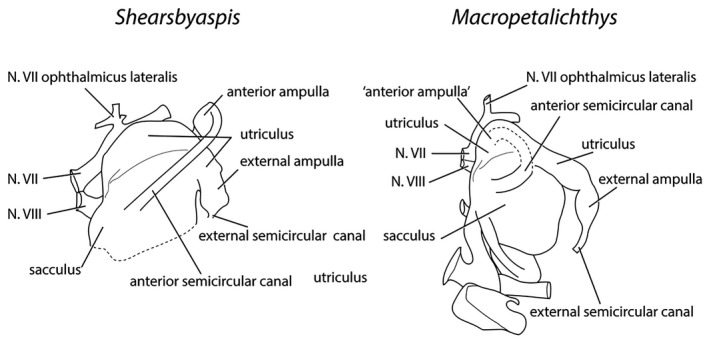
The morphology of the skeletal labyrinth of *Shearsbyaspis* (left) and *Macropetalichthys* (right) compared. *Macropetalichthys* is redrawn from Stensiö (1969) with original labelling. The ‘anterior ampulla’ is a doubtful interpretation (see text).

The anterior ampulla is situated very close to the posterior margin of the orbit (Figs 3, 4). The anterior semicircular canal crosses the sacculus from about the (presumed) centre of the dorsal side to the anterior ampulla at its anterolateral corner. The anterior ampulla is connected to the external ampulla by a ventrally flexed utriculus (Figs 3, 9, 10). The position of the anterior ampulla in *Shearsbyaspis* is very different from what has been reconstructed by Stensiö for *Macropetalichthys* (1963b, 1969), casting doubt on this reconstruction (Fig. 11). What was identified by Stensiö as the anterior ampulla is instead an anterolaterally placed utriculus, with the same position and morphology as in *Shearsbyaspis*. The anterior ampulla in *Macropetalichthys* is likely to have been in the same position as *Shearsbyaspis* but obscured by matrix or obliterated by preparation in the specimen studied by Stensiö (1690, text‐fig. 50A, 50B). Thus, it is likely that the anterior semicircular canal of *Macropetalichthys* traversed obliquely towards its anterolateral corner, rather than looping back medially (Fig. 11).

## Discussion

### Phylogenetic summary

**Figure 12:**
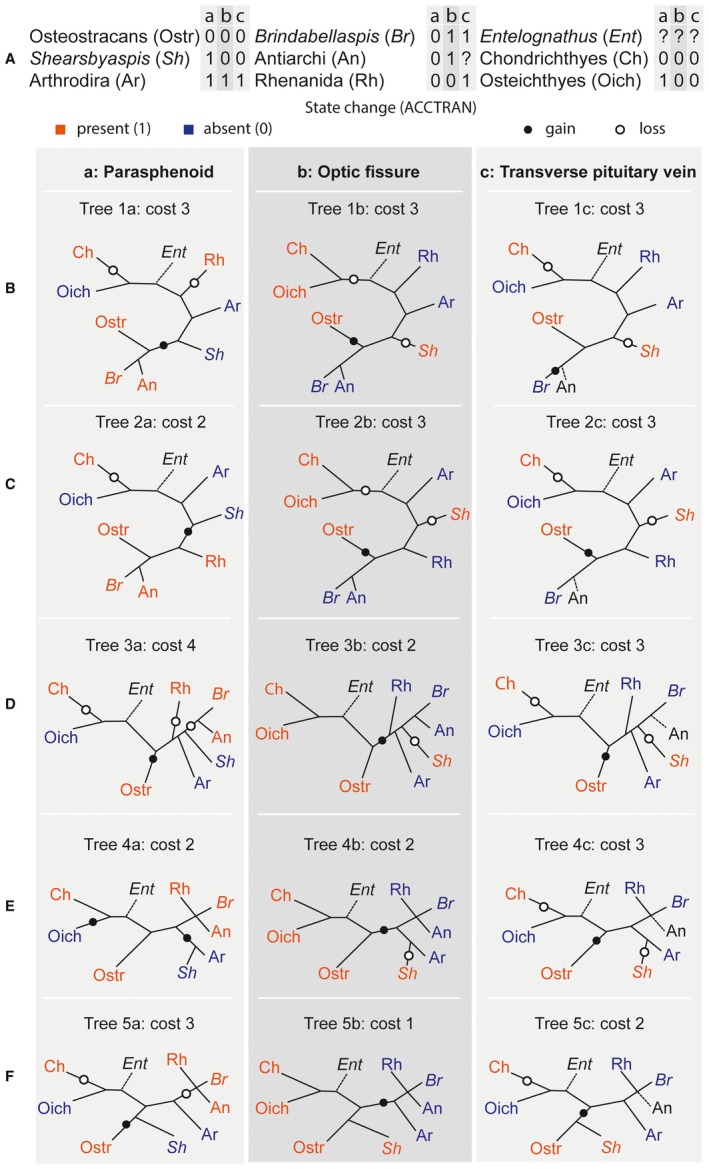
The distribution of the parasphenoid, the optic fissure and of the transverse pituitary vein on different unrooted topologies showing the evolutionary cost for each topology. A, data matrix with the scoring of the characters for each examined taxa. B, topology 1 represents the most parsimonious output for the distribution of: 1a, parasphenoid; 1b, optic fissure; 1c, transverse pituitary vein. C, topology 2 represents the character distribution with a paraphyletic Placodermi as found in Giles *et al*. (2015). D, topology 3 tests the result with a monophyletic Placodermi as found by King *et al*. (2016). E, topology 4 presents a monophyletic Placodermi with *Shearsbyaspis* resolved as sister to arthrodires. F, topology 5 presents a monophyletic Placodermi with *Shearsbyaspis* resolved as sister to the rest of the placoderms. Colour online.

The purpose of the present work is not to resolve placoderm relationships, but to add important new phylogenetic data and draw attention to problems with current, competing hypotheses. We are currently preparing a more inclusive analysis of placoderm relationships, but discuss here some of the implications of characters described in this work. We examine the compatibility of a limited, but important subset of phylogenetically informative characters bearing on the question of placoderm relationships. The unrooted trees in Figure 12 show several competing scenarios based on recently published hypotheses. Of the topologies considered, those with the lowest transformation cost are the two arrangements indicating the monophyly of a least a subset of placoderms. This agrees with King *et al*. (2016) and contrasts with previous, recent numerical hypotheses.

A revival of placoderm monophyly needs to account for the newly discovered ‘placoderm’ facial jaw skeletons from the Silurian of China: *Entelognathus* (Zhu *et al*. 2013) and *Qilinyu* (Zhu *et al*. 2016). Two competing hypotheses can account for this. One would place *Entelognathus* and *Qilinyu* outside of the main placoderm clade. Given the arthrodire‐like configuration of the braincase of *Entelognathus*, this new outgrouping could imply arthrodire‐like primitive skull architecture for placoderms as a whole. By consequence, many of the resemblances of ‘acanthothoracids’ and petalichthyids with jawless outgroups could be explained as being secondarily derived. The second hypothesis would place *Entelognathus* and *Qilinyu* as close sister groups of the gnathostome crown (as in current phylogenies). However, this could similarly imply an arthrodire‐like morphotype for not only placoderms, but also jaw‐bearing gnathostomes as a whole. The phylogenetic interpretation of petalichthyids in these competing scenarios (as either primarily or secondarily ‘osteostracan’ like) is central to reconstructing cranial transformations in early gnathostomes.

### Anatomical comparisons with the other early gnathostomes and their potential phylogenetic significance

#### Parasphenoid

Parasphenoid bones have previously only been convincingly observed in arthrodire placoderms and osteichthyans. Reports of a parasphenoid in *Bothriolepis* (Young 1986; Dennis‐Bryan 1995) are unsubstantiated. Its presence was only referred to in personal comments, and never formally described in spite of the large number of available specimens of this taxon. Dupret *et al*. (2017a) reported the presence of a parasphenoid in *Romundina*, although they argued that it does not seem to be formed of dermal bone so that the real nature of the material surrounding the hypophysial fenestra is unclear. A parasphenoid is probably present in *Kosoraspis* (Gross 1959). It is absent in other members of the ‘Acanthothoracida’. As the monophyly of this group has not been consistently recovered (e.g. Giles *et al*. 2015; King *et al*. 2016; Zhu *et al*. 2016), the phylogenetic interpretation of the parapshenoid in *Kosoraspis* is unclear.

Given the restricted distribution of parasphenoids, the lack of known petalichthyid examples, and hypotheses of placoderm paraphyly, the observation of a parasphenoid in *Shearsbyaspis* is surprising. Some caution must be exercised in making phylogenetic hypotheses about the parasphenoid in early vertebrates. Interpreting genuine absences can be problematic. In taxa lacking an interdigitating suture between the basicranium and the parasphenoid, the parasphenoid may easily become detached post mortem and therefore be unpreserved or unassociated with the braincase (Goujet 1984; Carr & Hlavin 2010; Zhu & Zhu 2013). However, we consider here the immediate phylogenetic implications of a parasphenoid in *Shearsbyaspis* given its unexpected observation and the fact that for many taxa (e.g. antiarchs) there is sufficient sampling to presume that this bone is absent. However, the presence of a parasphenoid in *Kosoraspis* (Gross 1959) might indicate that the distribution of this bone was more widespread in placoderms and not unique to petalichthyids and arthrodires.

The simplest interpretation for the parasphenoid character, if considered in isolation, would be the partitioning of *Shearsbyaspis*, arthrodires and osteichthyans together to the exclusion of all other taxa (Fig. 12B, tree 1a). Such a placement would imply at least some placoderms within the gnathostome crown group (as the bone is absent in chondrichthyans). We consider such a hypothesis to be unlikely based on present evidence (as it is not supported by any contemporary phylogenetic analyses). However, it cannot be overlooked simply because of this.

A second alternative accommodates a gnathostome stem group position for placoderms and places *Shearsbyaspis* together in a split with arthrodires (Fig. 12E, tree 4a). This would require only one additional step. Such a placement is consistent with earlier hypotheses suggesting close relationships of arthrodires and petalichthyids, although these were drawn under the presupposition of placoderm monophyly (Goujet & Young 1995, 2004). Indeed, the earliest known petalichthyids (quasipetalichthyids) (Zhu 1991; Pan *et al*. 2015) bear a strong external resemblance to early arthrodires, such as *Bryantolepis* (Elliott & Carr 2010) and *Wuttagoonaspis* (Ritchie 1973). This would be consistent with convergent similarities between osteostracan and macropetalichthyid neurocranial morphology.

#### Optic fissure

Several placoderm groups display nasal capsules separated from the rest of the braincase by a perichondrally fissure passing through the canal for the optic nerve canal. It completely subdivides the braincase into postethmoid and rhinocapsular portions. This fissure, termed the optic fissure, has been identified in arthrodires (Stensiö 1969; Young 1979; Goujet 1984), ‘acanthothoracids’ (Ørvig 1975; Young 1980; Olive *et al*. 2011), rhenanids (Stensiö 1950, 1969), and is inferred to have been present in antiarchs (Young 1984; Lukševičs 2001). In petalichthyids the nasal capsules are usually not preserved, suggesting the presence of separate cartilages containing the nasal capsules (Zhu 1991). This has been proposed as a possible placoderm synapomorphy by Brazeau & Friedman (2014). There is no such fissure observed in *Shearsbyaspis* or *Macropetalichthys* (Stensiö 1925, 1963a, b, 1969). Thus, the absence of a distinct optic fissure division separates petalichthyids from nearly all other placoderms (Fig. 12F, tree 5b).

However, a rhinocapsular division has been inferred in ‘quasipetalichthyids’, although it has not been directly observed (Zhu 1991; Pan *et al*. 2015). Furthermore, we observe a ridge inside the orbit of *Shearsbyaspis*, corresponding with the external signature of the partially closed fissure in *Brindabellaspis* (Young 1980, 1986). Furthermore, the division is apparently absent in eubrachythoracid arthrodires (Dennis‐Bryan & Miles 1979, 1983). The latter is probably a derived condition, but shows that the presence of a fissure is not necessarily universal among non‐petalichthyid placoderms. These considerations raise caution about interpreting the phylogenetic signal of this absence in petalichthyids and further data from ‘quasipetalichthyids’ in particular will help to resolve this problem.

#### Hypophyseal duct

In modern vertebrates, the hypophysis displays two very different morphologies. In cyclostomes, it projects anteriorly connecting with the nasal capsules. In crown‐group gnathostomes, it is independent from the nasal capsules and primitively projects posteriorly or posteroventrally as a duct. Fossil jawless fishes appear to exhibit the cyclostome condition, possessing an anteriorly‐oriented hypophysis, either connected with the nasal capsules, as in osteostracans (Janvier 1985), or as a separate anteroventrally‐oriented hypophyseal duct, as in the galeaspid *Shuyu* (Gai *et al*. 2011; Gai & Zhu 2012). In placoderms, the hypophyseal duct is projected either anteroventrally, as in *Romundina* (Dupret *et al*.2017a) or *Brindabellaspis* (Young 1980), or ventrally, as in arthrodires (Stensiö 1969; Goujet 1984). Dupret *et al*. (2017a) suggested that this represents a morphocline documenting the stepwise transformation of the hypophyseal duct from anteriorly directed to posteriorly directed. In this scenario, *Shearsbyaspis* could be interpreted as exhibiting a shared primitive state with jawless fishes and some placoderms (Fig. 12F). Alternatively, the conditions in either *Shearsbyaspis* or arthrodires could be secondarily derived, if petalichthyids are considered to be a proximate sister group of arthrodires (Fig. 12E).

#### Trigeminal–pituitary morphology and the posteroventral myodome

**Figure 13:**
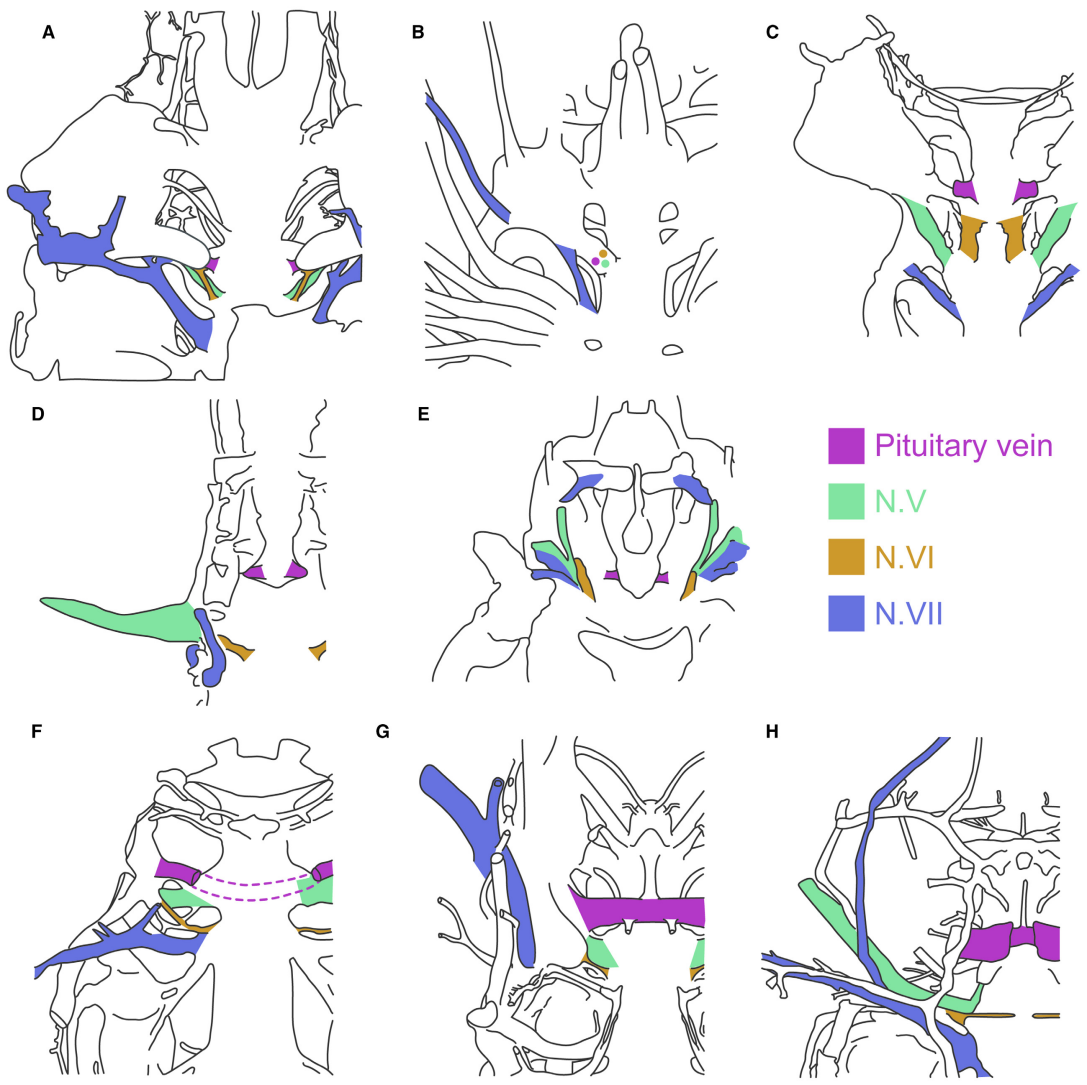
Endocranial cavities of several gnathostomes, in ventral view, highlighting the morphology of the trigeminal, abducens and facial nerves and of the pituitary vein. A, *Shearsbyaspis*, a petalichthyid placoderm. B, *Norselaspis*, an osteostracan (after Janvier 1981). C, *Shuyu*, a galeaspid (after Gai *et al*. 2011). D, *Cladodoides*, a chondrichthyan (after Maisey 2005). E, *Lawrenciella*, an osteichthyans (after Hamel & Poplin 2008). F, *Kujdanowiaspis*, an arthrodire placoderm (after Goujet 1984). G, *Brindabellaspis*, an ‘acanthothoracid’ placoderm (after Young 1980). H, *Romundina*, an ‘acanthothoracid’ placoderm (after Dupret *et al*. 2014). Not to scale.

The posteroventral myodome and associated canals in *Shearsbyaspis* and *Macropetalichthys* strongly resembles conditions in osteostracans. In these taxa, (Fig. 13A, B) the main trunk of the trigeminal and abducens nerves, and the pituitary vein canals, enter the orbit via the deep posteroventral myodome (Stensiö 1963b, 1969; Janvier 1981, 1985). The opening of these three canals deep within the posteroventral myodome is not observed in other placoderms (Young 1979; Goujet 1984; Young 1980; Olive *et al*. 2011; Dupret *et al*. 2017a) and crown gnathostomes (Gardiner 1984; Basden & Young 2001; Maisey 2005) (Fig. 13D–H).

In addition, petalichthyids, osteostracans and galeaspids (Gai *et al*. 2011) are distinct from non‐petalichthyid placoderms by having two separate canals for the pituitary vein (Fig. 13A–C). This is opposed to the condition in other placoderms (rhenanids: Stensiö 1969; *Brindabellaspis*: Young 1980; *Romundina*: Dupret *et al*. 2017a; and arthrodires: Stensiö 1969; Goujet 1984) where the pituitary canal is represented by a transverse canal spanning the width of the interorbital space (Fig. 13F–H). A fully enclosed, horizontal canal for the pituitary vein has been reported in the early osteichthyan *Ligulalepis* (Basden & Young 2001), but not in early chondrichthyans. This suggests that a single transverse pituitary vein canal may represent the primitive condition for crown gnathostomes.

The morphology of the pituitary vein and trigeminal nerve in association with the posteroventral myodome is parsimoniously interpreted as a shared primitive condition of osteostracans and macropetalichthyids (Fig. 12). However, we note that this phylogenetic arrangement would require the placement of macropetalichthyids as the sister group of other gnathostomes in which this part of the neurocranium is documented. This situation would conflict with existing phylogenetic hypotheses that place *Brindabellaspis* as a more distant relative of the gnathostome crown, relative to macropetalichthyids. This highlights some of the internal character conflicts arising from placoderm paraphyly.

#### Facial nerve, hyomandibular attachment and the similarities between placoderms and jawless fishes

Arguments for placoderm paraphyly are based in large part on the disposition of the facial nerve canals, hyomandibular articulation relative to the orbit, and the morphology of the braincase surrounding the orbit (Brazeau 2009; Brazeau & Friedman 2014). It has been argued by these authors that petalichthyids and *Brindabellaspis* resemble jawless osteostracans with respect to these characters. By contrast, arthrodires are interpreted as more closely resembling crown‐group gnathostomes (see Brazeau & Friedman 2014 for details). Recently, however, these characters were critically examined by King *et al*. (2016), who noted that they are morphologically related to the relative position of the jaws, and that some of these characters could therefore be expected to co‐vary. We agree with these assessments and add further details to the partitioning of these characters based on our examination of *Shearsbyaspis* and in light of recently published information on *Romundina* (Dupret *et al*. 2017a).

The character concerning the course of the facial nerve was removed from the matrix of King *et al*. (2016) on the grounds that it was redundant with respect to the position of the hyoid arch. However, we can show that it is not entirely redundant, though our new formulation does not necessarily corroborate recent hypotheses of placoderm paraphyly. Existing datasets, including that of King *et al*., divide total group gnathostomes into two character states: those with an anteriorly situated hyomandibular articulation (lateral or anterior to the posterior orbital wall) and those with an articulation situated posterior to the level of rear orbital wall. King *et al*. (2016) argued that the exposed course of the facial nerve within the orbit is correlated with the former state, an argument that follows logically from the relative position of the articulation and the associated nerve foramen. However, we note that in *Romundina*, the facial nerve division is exposed within the rear wall of the orbit, in spite of a posteriorly situated hyomandibular attachment. In effect, with respect to hyomandibular articulations and the course of the facial nerve, *Romundina* has been coded as resembling arthrodire and crown gnathostome conditions. By contrast, petalichthyids (i.e. *Macropetalichthys*) have been coded as resembling conditions in osteostracans and *Brindabellaspis*.

However, if we examine the respective morphologies in greater detail, and in light of the anatomy observed in *Shearsbyaspis*, we can demonstrate that these contrasts are incorrect. The course of the facial nerve in *Shearsbyaspis* resembles *Macropetalichthys*, where the hyomandibular branch traverses the rear wall of the orbit, before entering the transverse otic process and exiting next to the hyomandibular articulation. However, as in arthrodires and crown gnathostomes, the palatine branch does not enter the orbital space, nor does the common trunk of the facial nerve breach this wall. Instead, the palatine branch extends downward through the orbital floor, and exists from the palatal surface of the braincase, behind the anteroposterior level of the hypophysis. By contrast, the palatine branch in *Romundina* traverses anteriorly on the dorsal surface of the subocular shelf. Indeed, the division of the palatine and hyomandibular branches can be reasonably inferred to have occurred within the orbital cavity (Dupret *et al*. 2017a, text‐figs D–E). In this respect, the trunk of the facial nerve enters the orbital cavity, and *Romundina* resembles *Brindabellaspis*, despite the differences in the extension of the transverse otic process in these taxa.

We therefore argue that the morphology and division of the facial nerve has phylogenetic value, and that this can be shown to be (at least partially) independent of the position of the hyomandibula. However, the contrasts implied by examining these characters in further detail suggests closer similarities between petalichthyids and arthrodires than either share with *Brindabellaspis* or *Romundina*. We would therefore suggest inclusion of a modified version of this character that better accounts for the new knowledge from *Shearsbyaspis* and *Romundina*.

## Conclusion

We have presented the updated cranial morphology of *Shearsbyaspis oepiki* Young, 1985 based on computed tomography analysis. The new data presented here, augment our understanding of the morphology of petalichthyids. Given the centrality of this group to questions of placoderm relationships, our new data have shed light on the characters that have been the basis of arguments for placoderm paraphyly. We showed that while *Shearsbyaspis* has some interesting resemblances with osteostracans, in the relative position of the pituitary and trigeminal nerve foramina, and the absence of an optic fissure, it presents important characters (such as a parasphenoid) that stand in contrast with this. Additionally, the morphology of *Shearsbyaspis* invited critical reinvestigation of the diversity of facial nerve branching in early gnathostomes. We found that this variable is not strictly correlated with the position of the articulation of the hyomandibula on the braincase wall. However, accurate representation of its variation has not been presented in existing morphological datasets to date. Finally, the character compatibility examples we present raise questions about placoderm paraphyly on a character‐based level. Given the importance of this new data, we advocate that the best tests of placoderm relationships will come in the form of more inclusive analyses and better investigations of less well understood placoderm groups, such as petalichthyids. In particular, further knowledge of the so‐called ‘quasipetalichthyids’ (Liu 1973; Zhu 1991; Liu 1991; Pan *et al*. 2015) will add further information on the status of petalichthyids as a whole. These taxa share some significant features in common with petalichthyids that warrant consideration of their belonging in a clade together. However, quasipetalichthyids are both the earliest known petalichthyid relatives and bear strong resemblances to certain arthrodires (Zhu 1991; Pan *et al*. 2015). More information on the morphology of the skull of quasipetalichthyids could illuminate questions such as whether the optic fissure was lost in petalichthyids, or represents a shared primitive absence with non‐placoderms.

## References

[pala12345-bib-0001] Basden, A. M. and Young, G. C. 2001 A primitive actinopterygian neurocranium from the Early Devonian of southeastern Australia. Journal of Vertebrate Paleontology, 21 (4), 754–766.

[pala12345-bib-0002] Brazeau, M. D. 2009 The braincase and jaws of a Devonian acanthodian and modern gnathostome origins. Nature, 457, 305–308. 19148098 10.1038/nature07436

[pala12345-bib-0003] Brazeau, M. D. and Friedman, M. 2014 The characters of Palaeozoic jawed vertebrates. Zoological Journal of the Linnean Society, 170 (4), 779–821. 25750460 10.1111/zoj.12111 PMC4347021

[pala12345-bib-0004] Carr, R. K. and Hlavin, W. J. 2010 Two new species of *Dunkleosteus* Lehman, 1956, from the Ohio Shale Formation (U.S.A., Famennian) and the Kettle Point Formation (Canada, Upper Devonian), and a cladistic analysis of the Eubrachythoraci (Placodermi, Arthrodira). Zoological Journal of the Linnean Society, 159 (1), 195–222.

[pala12345-bib-0005] Castiello, M. and Brazeau, M. D. 2017 Shearsbyaspis parasphenoid (video). *Figshare* https://figshare.com/s/06c097d925ab4d121f4e

[pala12345-bib-0006] Coates, M. I. and Sequeira, S. E. K. 1998 The braincase of a primitive shark. Transactions of the Royal Society of Edinburgh: Earth Sciences, 89, 63–85.

[pala12345-bib-0007] Coates, M. I. and Sequeira, S. E. K. 2001 A new stethacanthid chondrichthyan from the Lower Carbonifeous of Bearsden, Scotland. Journal of Vertebrate Paleontology, 21, 438–459.

[pala12345-bib-0008] Davis, S. P. , Finarelli, J. A. and Coates, M. I. 2012 *Acanthodes* and shark‐like conditions in the last common ancestor of modern gnathostomes. Nature, 486, 247–250. 22699617 10.1038/nature11080

[pala12345-bib-0009] Denison, R. 1978 Placodermi *. In* Schultze H.‐P. (ed.) Handbook of paleoichthyology. Vol. 2 Gustav Fischer, 128 pp.

[pala12345-bib-0010] Dennis‐Bryan, K. 1995 Some comments on the placoderm parasphenoid. Bulletin du Muséum national d'Histoire naturelle C, 17 (1–4), 127–142.

[pala12345-bib-0011] Dennis‐Bryan, K. D. and Miles, R. S. 1979 Eubrachythoracid arthrodires with tubular rostral plates from Gogo, Western Australia. Zoological Journal of the Linnean Society, 67, 297–328.

[pala12345-bib-0012] Dennis‐Bryan, K. D. and Miles, R. S. 1983 Further eubrachythoracid arthrodires from Gogo, Western Australia. Zoological Journal of the Linnean Society, 77, 145–173.

[pala12345-bib-0013] Dupret, V. 2010 Revision of the genus *Kujdanowiaspis* Stensiö, 1942 (Placodermi, Arthrodira, Actinolepida) from the Lower Devonian of Podolia (Ukraine). Geodiversitas, 32 (1), 5–63.

[pala12345-bib-0014] Dupret, V. , Zhu, M. I. N. and Wang, J. Q. 2009 The morphology of *Yujiangolepis liujingensis* (Placodermi, Arthrodira) from the Pragian of Guangxi (south China) and its phylogenetic significance. Zoological Journal of the Linnean Society, 157 (1), 70–82.

[pala12345-bib-0015] Dupret, V. , Sanchez, S. , Goujet, D. , Tafforeau, P. and Ahlberg, P. E. 2014 A primitive placoderm sheds light on the origin of the jawed vertebrate face. Nature, 507, 500–503. 24522530 10.1038/nature12980

[pala12345-bib-0016] Dupret, V. , Sanchez, S. , Goujet, D. and Ahlberg, P. E. 2017a The internal cranial anatomy of *Romundina stellina* Ørvig, 1975 (Vertebrata, Placodermi, Acanthothoraci) and the origin of jawed vertebrates – anatomical atlas of a primitive gnathostome. PLoS One, 12 (2), e0171241. 28170434 10.1371/journal.pone.0171241 PMC5295682

[pala12345-bib-0017] Dupret, V. , Zhu, M. and Wang, J. Q. 2017b Redescription of *Szelepis* Liu, 1981 (Placodermi, Arthrodira) from the Lower Devonian of China. Journal of Vertebrate Paleontology, 37, e1312422.

[pala12345-bib-0018] Eastman, C. R. 1898 Some new points in dinichthyid osteology. The American Naturalist, 32, 747–768.

[pala12345-bib-0019] Elliott, D. K. and Carr, R. K. 2010 A new species of *Bryantolepis* Camp, Welles, and Green, 1949 (Placodermi, Arthrodira) from the Early Devonian Water Canyon Formation of Northern Utah and Southern Idaho, with comments on the endocranium. Kirtlandia, 57, 22–35.

[pala12345-bib-0020] Forey, P. L. and Gardiner, B. G. 1986 Observations on *Ctenurella* (Ptyctodontida) and the classification of placoderm fishes. Zoological Journal of the Linnean Society, 86, 43–74.

[pala12345-bib-0021] Friedman, M. 2007 *Styloichthys* as the oldest coelacanth: implications for early osteichthyan interrelationships. Journal of Systematic Palaeontology, 5 (3), 289–343.

[pala12345-bib-0022] Gai, Z. and Zhu, M. 2012 The origin of the vertebrate jaw: Intersection between developmental biology‐based model and fossil evidence. Chinese Science Bulletin, 57 (30), 3819–3828.

[pala12345-bib-0023] Gai, Z. , Donoghue, P. C. J. , Zhu, M. , Janvier, P. and Stampanoni, M. 2011 Fossil jawless fish from China foreshadows early jawed vertebrate anatomy. Nature, 476, 324–327. 21850106 10.1038/nature10276

[pala12345-bib-0024] Gardiner, B. G. 1984 The relationships of the palaeoniscid fishes, a review based on new specimens of *Mimia* and *Moythomasia* from the Upper Devonian of Western Australia. Bulletin of the British Museum (Natural History): Geology, 37, 173–428.

[pala12345-bib-0025] Garwood, R. and Dunlop, J. 2014 The walking dead: Blender as a tool for paleontologists with a case study on extinct arachnids. Journal of Paleontology, 88, 735–746.

[pala12345-bib-0026] Giles, S. , Friedman, M. and Brazeau, M. D. 2015 Osteichthyan‐like cranial conditions in an Early Devonian stem gnathostome. Nature, 520, 82–85. 25581798 10.1038/nature14065 PMC5536226

[pala12345-bib-0027] Goujet, D. 1984 Les Poissons placodermes du Spitsberg. Arthrodires Dolichothoraci de la Formation de Wood Bay (Dévonien inférieur). Cahiers de Paléontologie (Vertébrés) Éditions du CNRS, Paris.

[pala12345-bib-0028] Goujet, D. 2001 Placoderms and basal gnathostome apomorphies 209–222. *In* Ahlberg P. E. (ed.) Major events in early vertebrate evolution: palaeontology, phylogeny, genetics and development. Systematics Association Special Volume 61, Taylor & Francis.

[pala12345-bib-0029] Goujet, D. and Young, G. C. 1995 Interrelationships of placoderms revisited. Geobios Mémoire Spécial, 19, 89–95.

[pala12345-bib-0030] Goujet, D. and Young, G. C. 2004 Placoderm anatomy and phylogeny: new insights *. In* Arratia G., Wilson M. V. H. and Cloutier R. (eds). Recent advances in the origin and early radiation of vertebrates. Friedrich Pfeil, 109–126.

[pala12345-bib-0031] Gross, W. 1959 Arthrodiren aus dem Obersilur der Prager Mulde. Palaeontolographica, 113 (A), 1–35.

[pala12345-bib-0032] Hamel, M.‐H. and Poplin, C. 2008 The braincase anatomy of *Lawrenciella schaefferi*, actinopterygian from the Upper Carboniferous of Kansas (U.S.A.). Journal of Vertebrate Paleontology, 28 (4), 989–1006.

[pala12345-bib-0033] Janvier, P. 1975 Les yeux des Cyclostomes fossiles et le problème de l'origine des Myxinoïdes. Acta Zoologica, 56 (1), 1–9.

[pala12345-bib-0034] Janvier, P. 1981 *Norselaspis glacialis* n.g., n.sp. et les relations phylogénétiques entre les Kiaeraspidiens (Osteostraci) du Dévonien inférieur du Spitsberg. Palaeovertebrata, 11, 19–131.

[pala12345-bib-0035] Janvier, P. 1985 Les Cephalaspides du Spitsberg. Anatomie, phylogénie et systematique des Ostéostracés siluro‐dévoniens. Révision des Osteostracés de la Formation de Wood Bay (Dévonien inférieur du Spitsberg). Cahiers de Paléontologie, Centre National de la Recherche Scientifique, Paris, 244 pp.

[pala12345-bib-0036] Janvier, P. 1996a Early vertebrates. Oxford Monographs on Geology & Geophysics, Clarendon Press.

[pala12345-bib-0037] Janvier, P. 1996b The dawn of the vertebrates: characters versus common ascent in the rise of current vertebrate phylogenies. Palaeontology, 39 (2), 259–287.

[pala12345-bib-0038] King, B. , Qiao, T. , Lee, M. S. Y. , Zhu, M. and Long, J. A. 2016 Bayesian morphological clock methods resurrect placoderm monophyly and reveal rapid early evolution in jawed vertebrates. Systematic Biology, 66, 499–516. 10.1093/sysbio/syw107 27920231

[pala12345-bib-0039] Liu, Y. H. 1973 On the new forms of Polybranchiaspiformes and Petalichthyida from Devonian of South‐west China. Vertebrata PalAsiatica, 11, 132–143.

[pala12345-bib-0040] Liu, Y. H. 1991 On a new petalichthyid, *Eurycaraspis incilis* gen. et sp. nov., from the Middle Devonian of Zhanyi, Yunnan *. In* Chang M., Liu Y. and Zhang G. (eds). EarIy vertebrates and related problems of evolutionary biology. Science Press, Beijing, 139 pp.

[pala12345-bib-0041] Long, J. A. , Mark‐Kurik, E. , Johanson, Z. , Lee, M. S. , Young, G. C. , Min, Z. , Ahlberg, P. E. , Newman, M. , Jones, R. , den Blaauwen, J. , *et al* 2015 Copulation in antiarch placoderms and the origin of gnathostome internal fertilization. Nature, 517, 196–199. 25327249 10.1038/nature13825

[pala12345-bib-0042] Lukševičs, E. 2001 The orbito‐nasal area of *Asterolepis ornata*, a Middle Devonian placoderm fish. Journal of Vertebrate Paleontology, 21 (4), 687–692.

[pala12345-bib-0043] Maisey, J. G. 2005 Braincase of the Upper Devonian shark *Cladodoides wildungensis* (Chondrichthyes, Elasmobranchii), with observations on the braincase in early chondrichthyans. Bulletin of the American Museum of Natural History, 228, 1–103.

[pala12345-bib-0044] Maisey, J. G. 2007 The braincase in Paleozoic symmoriiform and cladoselachian sharks. Bulletin of the American Museum of Natural History, 307, 1–122.

[pala12345-bib-0045] Miles, R. 1967 Observations on the ptyctodont fish, *Rhamphodopsis* Watson. Journal of the Linnean Society of London, Zoology, 47, 99–120.

[pala12345-bib-0046] Olive, S. , Goujet, D. , Lelièvre, H. and Janjou, D. 2011 A new Placoderm fish (Acanthothoraci) from the Early Devonian Jauf Formation (Saudi Arabia). Geodiversitas, 33 (3), 393–409.

[pala12345-bib-0047] Ørvig, T. 1957 Notes on some Paleozoic lower vertebrates from Spitsbergen and North America. Norsk Geologisk Tiddskrift, 37, 285–353.

[pala12345-bib-0048] Ørvig, T. 1975 Description, with special reference to the dermal skeleton, of a new radotinid arthrodire from the Gedinnian of Arctic Canada. Colloques internationaux du Centre national de la Recherche scientifique, 218, 43–71.

[pala12345-bib-0049] P'an, K. and Wang, S. T. 1978 Devonian Agnatha and Pisces of South China *In* Institute of Mineral Resources, Chinese Academy of Geological Sciences (eds). Symposium on the Devonian System of South China. Geological Publishing House Press, Beijing, 298–333.

[pala12345-bib-0050] Pan, Z. , Zhu, M. , Zhu, Y. and Jia, L. 2015 A new petalichthyid placoderm from the Early Devonian of Yunnan, China. Comptes Rendus Palevol, 14 (2), 125–137.

[pala12345-bib-0051] Ritchie, A. 1973 *Wuttagoonaspis* gen. nov., an unusual arthrodire from the Devonian of Western New South Wales, Australia. Palaeontographica, 143A, 58–72.

[pala12345-bib-0052] Stensiö, E. A. 1925 On the head of the macropetalichthyids with certain remarks on the head of other arthrodires. Publications of the Field Museum of Natural History, Geological Series, 4, 87–197.

[pala12345-bib-0053] Stensiö, E. A. 1950 La cavite labyrinthique, l'ossification sclerotique et l'orbite de *Jagorina* . Colloques internationaux du Centre national de la Recherche scientifique, 21, 9–41.

[pala12345-bib-0054] Stensiö, E. A. 1963a Anatomical studies on the arthrodiran head. 1. Preface, geological and geographical distribution, and organisation of the arthrodires, the anatomy of the head in the Dolichothoraci, Coccosteomorphi and Pachyosteomorphi. Kungliga Svenska Vetenskapsakademiens Handlingar, 9, 1–419.

[pala12345-bib-0055] Stensiö, E. A. 1963b The brain and the cranial nerves in fossil, lower craniate vertebrates. Skriflter Ulgitt Av Det Norske Videnskaps‐Akademi, 13, 1–120.

[pala12345-bib-0056] Stensiö, E. A. 1969 Elasmobranchiomorphi Placodermata Arthrodires 71–692. *In* Piveteau J. (ed.) Traité de Paléontologie 4. Masson, Paris.

[pala12345-bib-0057] Trinajstic, K. , Long, J. A. , Johanson, Z. , Young, G. C. and Senden, T. 2012 New morphological information on the ptyctodontid fishes (Placodermi, Ptyctodontida) from Western Australia. Journal of Vertebrate Paleontology, 32 (4), 757–780.

[pala12345-bib-0058] Woodward, A. S. 1941 IX. The head shield of a new Macropetalichthyid fish (*Notopetalichthys hillsi*, gen. et sp. nov.) from the Middle Devonian of Australia. Annals & Magazine of Natural History, Series 11, 8 (44), 91–96.

[pala12345-bib-0059] Young, G. 1978 A new Early Devonian petalichthyid fish from the Taemas/Wee Jasper region of New South Wales. Alcheringa, 2 (2), 103–116.

[pala12345-bib-0060] Young, G. 1979 New information on the structure and relationships of *Buchanosteus* (Placodermi: Euarthrodira) from the Early Devonian of New South Wales. Zoological Journal of the Linnean Society, 66 (4), 309–352.

[pala12345-bib-0061] Young, G. C. 1980 A new Early Devonian placoderm from New South Wales, Australia, with a discussion of placoderm phylogeny. Palaeontographica, 167 (A), 10–76.

[pala12345-bib-0062] Young, G. 1984 Reconstruction of the jaws and braincase in the Devonian placoderm fish *Bothriolepis* . Palaeontology, 27 (3), 635–661.

[pala12345-bib-0063] Young, G. 1985 Further petalichthyid remains (Placoderm fishes, Early Devonian) from the Taemas‐Wee Jasper region, New South Wales. Journal of Australian Geology & Geophysics, 9, 121–131.

[pala12345-bib-0064] Young, G. C. 1986 The relationships of placoderm fishes. Zoological Journal of the Linnean Society, 88 (1), 1–57.

[pala12345-bib-0065] Young, G. C. 2008 Number and arrangement of extraocular muscles in primitive gnathostomes: evidence from extinct placoderm fishes. Biology Letters, 4 (1), 110–114. 18077236 10.1098/rsbl.2007.0545 PMC2413266

[pala12345-bib-0066] Zhu, M. 1991 New information on *Diandongpetalichthys* (Placodermi: Petalichthyida) *. In* Chang M.‐M., Liu Y.‐H. and Zhang G.‐R. (eds). EarIy vertebrates and related problems of evolutionary biology. Science Press, Beijing, 179 pp.

[pala12345-bib-0067] Zhu, M. 2000 Catalogue of Devonian vertebrates in China, with notes on bio‐events 373–390. *In* Blieck A. and Turner S. (eds). Palaeozoic vertebrate biochronology and global marine/non‐marine correlation. Final Report of IGCP 328 (1991–1996). Courier Forschungsinstitut Senckenberg, 223.

[pala12345-bib-0068] Zhu, M. and Schultze, H.‐P. 1997 The oldest sarcopterygian fish. Lethaia, 30, 293–304.

[pala12345-bib-0069] Zhu, M. and Wang, J. 1996 A new macropetalichthyid from China, with special reference to the historical zoogeography of the Macropetalichthyidae (Placodermi). Vertebrata PalAsiatica, 10 (34), 253–268.

[pala12345-bib-0070] Zhu, Y.‐A. and Zhu, M. 2013 A redescription of *Kiangyousteus yohii* (Arthrodira: Eubrachythoraci) from the Middle Devonian of China, with remarks on the systematics of the Eubrachythoraci. Zoological Journal of the Linnean Society, 169 (4), 798–819.

[pala12345-bib-0071] Zhu, M. , Yu, X. and Janvier, P. 1999 A primitive fossil fish sheds light on the origin of bony fishes. Nature, 397, 607–610.

[pala12345-bib-0072] Zhu, M. , Yu, X. , Wang, W. , Zhao, W. and Jia, L. 2006 A primitive fish provides key characters bearing on deep osteichthyan phylogeny. Nature, 441, 77–80. 16672968 10.1038/nature04563

[pala12345-bib-0073] Zhu, M. , Zhao, W. , Jia, J. , Lu, J. , Qiao, T. and Qu, Q. 2009 The oldest articulated osteichthyan reveals mosaic gnathostome characters. Nature, 458, 469–474. 19325627 10.1038/nature07855

[pala12345-bib-0074] Zhu, M. , Wang, J. Q. and Wang, S. T. 2010 A new antarctaspid arthrodire (placoderm fish) from the Lower Devonian of Guangxi, China. Vertebrata PalAsiatica, 48 (2), 100–110.

[pala12345-bib-0075] Zhu, M. , Yu, X. , Ahlberg, P. E. , Choo, B. , Lu, J. , Qiao, T. , Qu, Q. , Zhao, W. , Jia, L. , Blom, H. and Zhu, Y. 2013 A Silurian placoderm with osteichthyan‐like marginal jaw bones. Nature, 502, 188–193. 24067611 10.1038/nature12617

[pala12345-bib-0076] Zhu, M. , Ahlberg, P. E. , Pan, Z. , Zhu, Y. , Qiao, T. , Zhao, W. , Jia, L. and Lu, J. 2016 A Silurian maxillate placoderm illuminates jaw evolution. Science, 354 (6310), 334–336. 27846567 10.1126/science.aah3764

